# Genome biogeography reveals the intraspecific spread of adaptive mutations for a complex trait

**DOI:** 10.1111/mec.13914

**Published:** 2016-11-30

**Authors:** Jill K. Olofsson, Matheus Bianconi, Guillaume Besnard, Luke T. Dunning, Marjorie R. Lundgren, Helene Holota, Maria S. Vorontsova, Oriane Hidalgo, Ilia J. Leitch, Patrik Nosil, Colin P. Osborne, Pascal‐Antoine Christin

**Affiliations:** ^1^ Department of Animal and Plant Sciences University of Sheffield Western Bank Sheffield S10 2TN UK; ^2^ Laboratoire Évolution & Diversité Biologique (EDB UMR5174) Université de Toulouse CNRS, ENSFEA, UPS 118 route de Narbonne F‐31062 Toulouse France; ^3^ Royal Botanic Gardens Kew Richmond Surrey TW9 3AB UK

**Keywords:** adaptation, C_4_ photosynthesis, gene flow, lateral gene transfer, phylogeography

## Abstract

Physiological novelties are often studied at macro‐evolutionary scales such that their micro‐evolutionary origins remain poorly understood. Here, we test the hypothesis that key components of a complex trait can evolve in isolation and later be combined by gene flow. We use C_4_ photosynthesis as a study system, a derived physiology that increases plant productivity in warm, dry conditions. The grass *Alloteropsis semialata* includes C_4_ and non‐C_4_ genotypes, with some populations using laterally acquired C_4_‐adaptive loci, providing an outstanding system to track the spread of novel adaptive mutations. Using genome data from C_4_ and non‐C_4_ *A. semialata* individuals spanning the species’ range, we infer and date past migrations of different parts of the genome. Our results show that photosynthetic types initially diverged in isolated populations, where key C_4_ components were acquired. However, rare but recurrent subsequent gene flow allowed the spread of adaptive loci across genetic pools. Indeed, laterally acquired genes for key C_4_ functions were rapidly passed between populations with otherwise distinct genomic backgrounds. Thus, our intraspecific study of C_4_‐related genomic variation indicates that components of adaptive traits can evolve separately and later be combined through secondary gene flow, leading to the assembly and optimization of evolutionary innovations.

## Introduction

Over evolutionary time, living organisms have been able to colonize almost every possible environment, often via the acquisition of novel adaptations. While impressive changes can be observed across phyla, adaptive evolution by natural selection occurs within populations (e.g. Geber & Griffen 2003; Morjan & Rieseberg 2004). For most complex adaptive novelties, the intraspecific dynamics that lead to their progressive emergence are poorly understood. Indeed, if novel complex traits gain their function only when multiple anatomical and/or biochemical components work together, the order of acquisition of such components raises intriguing questions (Meléndez‐Hevia *et al*. 1996; Lenski *et al*. 2003). One possibility is that the acquisition of one key component is sufficient to trigger a novel trait (e.g. Ourisson & Nakatani 1994), allowing the subsequent selection of novel mutations for the other components in the genetic pool that fixed the first component. The alternative would assume that components accumulate independently of each other in isolated populations and are later assembled by secondary gene flow and subsequent selection to form the complex trait (Morjan & Rieseberg 2004; Leinonen *et al*. 2006; Hufford *et al*. 2013; Ellstrand 2014; Miller *et al*. 2014). Differentiating these scenarios requires the inference of the order of mutations for a novel complex trait, as well as their past spread throughout the history of divergence, migration and secondary gene flow in one or several related species. Such investigations must rely on study systems in which variation in an adaptive complex trait, and its underlying genomic basis, can be traced back through time.

C_4_ photosynthesis is a physiological state, present in ~3% of plant species (Sage 2016), which results from the co‐ordinated action of multiple enzymes and anatomical components (Hatch 1987; Christin & Osborne 2014). C_4_ biochemistry relies on well‐characterized enzymes that also exist in non‐C_4_ plants, but with altered abundance, cellular and subcellular localization, regulation and kinetics (Kanai & Edwards 1999). The main effect of C_4_ photosynthesis is an increase in CO_2_ concentration at the place of its fixation by the enzyme Rubisco in the Calvin–Benson cycle (von Caemmerer & Furbank 2003). This is advantageous in conditions that restrict CO_2_ availability, especially in warm and arid environments under the low‐CO_2_ atmosphere that has prevailed for the last 30 million years (Sage *et al*. 2012). C_4_ plants consequently dominate most open biomes in tropical and subtropical regions, where they achieve high growth rates and large biomass (Griffith *et al*. 2015; Atkinson *et al*. 2016). Despite its apparent complexity, C_4_ photosynthesis evolved more than 60 times independently over the ancestral C_3_ type (Sage *et al*. 2011), and evolutionary transitions were facilitated by the existence of anatomical and genetic enablers in some groups of plants (Christin *et al*. 2013b, 2015). However, the micro‐evolutionary history of photosynthetic transitions is yet to be addressed.

Most C_4_ lineages evolved this photosynthetic system millions of years ago, so that the initial changes linked to C_4_ evolution remain obscured (Christin & Osborne 2014). In a couple of groups, closely related species present a spectrum of more or less complete C_4_ traits, which is interpreted as the footprint of the gradual evolution of C_4_ (e.g. McKown *et al*. 2005; Christin *et al*. 2011; Fisher *et al*. 2015). These groups provide powerful systems to reconstruct the order of changes during the transition to C_4_ photosynthesis (e.g. McKown & Dengler 2007; Heckmann *et al*. 2013; Williams *et al*. 2013). However, the presumed lack of gene flow among these related species impedes testing hypotheses about the importance of secondary gene flow mixing mutations that were fixed in isolated populations. So far, the presence of genotypes with different photosynthetic types has been reported in only one taxon, the grass *Alloteropsis semialata*.

*Alloteropsis semialata* includes C_3_ and C_4_ individuals (Ellis 1974), and a recent study further described individuals with only some of the C_4_ anatomical and biochemical components, which allow a weak C_4_ cycle (i.e. C_3_–C_4_ intermediates; Lundgren *et al*. 2016). Other species in this genus, *Alloteropsis angusta*,* Alloteropsis cimicina*,* Alloteropsis paniculata* and *Alloteropsis papillosa*, are C_4_, but perform the C_4_ cycle using different enzymes and leaf tissues than *A. semialata*, which points to independent realizations of the C_4_ phenotype (Christin *et al*. 2010). Analyses of genes for key C_4_ enzymes in a handful of accessions have revealed that some populations of *A. semialata* carry C_4_ genes that have been laterally acquired from distant C_4_ relatives (Christin *et al*. 2012). The laterally acquired genes include one for phosphoenolpyruvate carboxykinase (*pck*) and three different copies for phosphoenolpyruvate carboxylase (*ppc*). These laterally acquired genes are integrated into the C_4_ cycle of some extant accessions of *Alloteropsis* (Christin *et al*. 2012, 2013a), but genes for other C_4_ enzymes have been transmitted following the species tree (vertically inherited), and gained their C_4_ function via novel mutations (Christin *et al*. 2013a). Some C_4_ *Alloteropsis* populations presumably still use the vertically inherited *ppc* and *pck* homologs for their C_4_ cycles. However, the laterally acquired *ppc* and *pck* copies spent millions of years in other C_4_ species, where they acquired adaptive mutations that likely increased their fit for the C_4_ function before their transfer (Christin *et al*. 2012). The potential adaptive value of the laterally acquired genes, as well as their restriction to some C_4_ populations, provides a tractable system to elucidate gene movements that led to the emergence and strengthening of the complex C_4_ adaptive trait. However, the geographical distributions and frequencies of these laterally acquired genes are still poorly understood, and the genome history of *A. semialata* remains largely unexplored.

**Figure 1:**
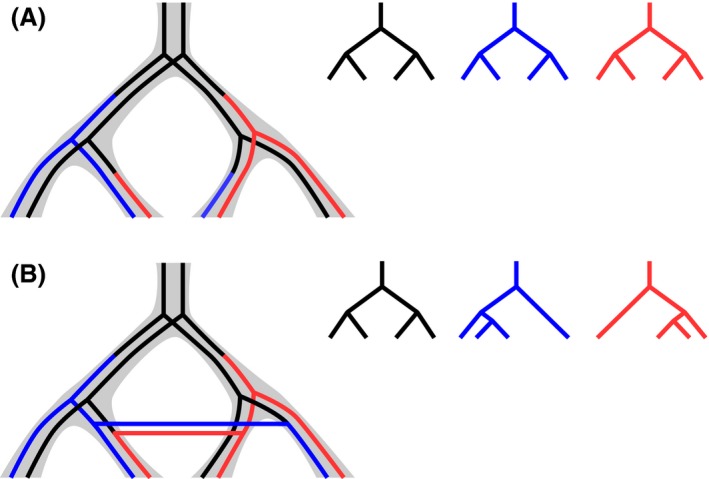
Competing scenarios for the assembly of a complex trait. (A) The trait is assembled by sequential fixation of mutations within each genetic pool. (B) Mutations that were fixed in isolation are later assembled via secondary gene flow. The species tree is outlined by thick grey branches, and coloured branches indicate novel mutations on individual genes. Individual gene trees are drawn on the right. In scenario A, the histories of adaptive mutations correspond to the history of the rest of the genome and all gene trees are concordant, while in scenario B, the histories of the adaptive mutations differ from that of the rest of the genome, with gene trees that do not match the species tree.

In this study, we obtain low‐coverage whole‐genome sequencing data from *A. semialata* individuals spread across the species’ geographical range and differing in photosynthetic type. We use the data to first infer the history of isolation and secondary contact, and then to track the acquisition and spread of the laterally acquired genes. This biogeographic framework allows us to test whether the C_4_ complex trait was assembled via the sequential fixation of novel mutations within each isolated gene pool or via gene flow combining mutations that had been fixed in distinct gene pools (Fig. 1). In the first scenario, the history of C_4_‐adaptive mutations, represented by the laterally acquired genes, would correspond to the sequence of migration and isolation of populations and largely match the history of the rest of the genome (Fig. 1A). In the second scenario, the history of C_4_‐adaptive mutations would differ from that of the rest of the genome, their selection‐driven spread across genetic lineages resulting in more recent coalescence times and gene topologies that differ from the species topology (Fig. 1B). This first intraspecific spatial genomic analysis of key components of the C_4_ complex trait opens new avenues to understand the micro‐evolutionary processes that led to macro‐evolutionary innovations.

## Material and methods

### Sampling, sequencing and genome sizing

A low‐coverage whole‐genome sequencing approach (genome skimming) was used to reconstruct the genome history of *Alloteropsis*. This approach has become increasingly attractive for inferring population parameters (e.g. Buerkle & Gompert 2013; Fumagalli *et al*. 2013) and for studying complex traits (Li *et al*. 2011). It also allows de novo assembly of high copy number regions of the genome, such as organelle genomes (Besnard *et al*. 2014; Dodsworth 2015), and can be applied to samples of limited quality and quantity, such as herbarium or museum collections (Besnard *et al*. 2014). Genome‐skimming data for eleven *Alloteropsis semialata* individuals, and one of each of the congeneric *Alloteropsis cimicina* and *Alloteropsis angusta*, were retrieved from a previous study that used them to assemble chloroplast genomes (Table S1, Supporting information; Lundgren *et al*. 2015, 2016). The photosynthetic type of these samples has been determined previously, and they encompass non‐C_4_ individuals with and without a weak C_4_ cycle, as well as multiple C_4_ accessions (Table S1, Supporting information; Lundgren *et al*. 2015, 2016). An additional eight *Alloteropsis* accessions were sampled here to increase the resolution of genome biogeography for the group (Table S1, Supporting information). These include one accession from each of the congeneric species *Alloteropsis paniculata* and *A. angusta*, and six additional *A. semialata* individuals. These samples were selected to increase the plastid and photosynthetic diversity, with a special focus on the Zambezian biogeographic region (spanning Tanzania, Zambia and the Democratic Republic of Congo – DRC; Linder *et al*. 2012; Table S1, Supporting information), where the majority of the chloroplast and photosynthetic diversities are found (Lundgren *et al*. 2015, 2016). Three of the newly sequenced *A. semialata* accessions (‘DRC3’, ‘TAN3’ and ‘KEN1’) were previously characterized with stable carbon isotopes (Lundgren *et al*. 2015), which can distinguish plants grown using mainly C_4_ photosynthesis from those that acquired a significant portion of their carbon via the ancestral C_3_ cycle, whether or not it is complemented by a weak C_4_ cycle (Smith & Brown 1973; Cerling *et al*. 1997). One of these three accessions (‘TAN3’) is isotopically intermediate, indicating that a strong C_4_ cycle occurs, but that some atmospheric carbon is still fixed directly by the C_3_ cycle (Peisker 1986; Monson *et al*. 1988). For four of the new samples, carbon isotopes were measured on a leaf fragment as previously described (Lundgren *et al*. 2015), which revealed that all of them had carbon isotope values within the C_4_ range (Table S1, Supporting information).

DNA was extracted, quality checked and sequenced as described in Lundgren *et al*. (2015), except that the DNA of these accessions was not sonicated prior to the library preparation due to the high degree of DNA degradation in these herbarium specimens. Each sample was individually barcoded and pooled with 23 other samples (from the same or unrelated projects) before paired‐end sequencing (100–150 bp) on one Illumina lane (HiSeq‐2500 or HiSeq‐3000) at the Genopole platform of Toulouse or at the Genoscope platform of Evry (only *A. paniculata*; Table S1, Supporting information). The final data set consisted of sequence data for a total of 21 individuals, sequenced in six different runs (Table S1, Supporting information).

The genome size was estimated for accessions for which live material was available by flow cytometry following the one‐step protocol of Doležel *et al*. (2007) with minor modifications as described in Clark *et al*. (2016). We selected *Oryza sativa* IR36 (2C = 1 pg; Bennett & Smith 1991) and the Ebihara buffer (Ebihara *et al*. 2005) as the most appropriate internal standard and nuclei isolation buffer for all but one accessions (Table S1, Supporting information). For the ‘RSA3’ accession, whose C‐value was estimated to be about three time larger than other accessions, we used the *Pisum sativum* ‘Ctirad’ standard (2C = 9.09 pg; Doležel *et al*. 1992) and the GPB buffer (Loureiro *et al*. 2007), supplemented with 3% of PVP.

### Assembly and analyses of chloroplast genomes

Complete chloroplast genomes were de novo assembled for the newly sequenced individuals using the genome walking method described in Lundgren *et al*. (2015). The newly generated chloroplast genomes were manually aligned with those already available, and a time‐calibrated phylogenetic tree was inferred with beast v. 1.5.4 (Drummond & Rambaut 2007), as described in Lundgren *et al*. (2015). Monophyly of the outgroup (*A. cimicina* + *A. paniculata*) and the ingroup (*A. angusta* + *A. semialata*) was enforced to root the phylogeny, which is consistent with all previous analyses (Ibrahim *et al*. 2009; Christin *et al*. 2012; GPWGII 2012; Lundgren *et al*. 2015). The root of the tree was fixed to 11 Ma (as found by Lundgren *et al*. 2015), which was achieved with a normal distribution of mean of 11 and standard deviation of 0.0001. Two different analyses were run for 20 000 000 generations, sampling a tree every 1000 generations. After checking the convergence of the runs in tracer v. 1.5.0 (Drummond & Rambaut 2007), the burn‐in period was set to 2 000 000 generations, and the maximum credibility tree was identified from the trees sampled after the burn‐in period in both analyses, mapping median ages on nodes.

### Genotyping across the nuclear genome

A reference genome for *Alloteropsis* is currently lacking. However, the grass *Setaria italica* (comment name: Foxtail millet) belongs to the same tribe as *Alloteropsis* (Paniceae) and has a well‐assembled reference genome (jgiv2.0.27; Bennetzen *et al*. 2012). *Setaria* and *Alloteropsis* diverged approximately 20 Ma (Christin *et al*. 2012), a time that is sufficient for a complete turnover of noncoding sequences (Ammiraju *et al*. 2008). However, reads corresponding to coding regions across the genome can still be reliably mapped (see Results).

Raw sequencing reads were quality filtered using the ngs qc toolkit v. 2.3.3 (Patel & Jain 2012). Reads with more than 20% of the bases having a quality score below Q20 and reads with ambiguous bases were removed. Furthermore, low‐quality bases (<Q20) were trimmed from the 3’ end of the remaining reads. The filtered reads were mapped to the *Setaria* reference genome, using bowtie2 v. 2.2.3 (Langmead *et al*. 2009). Raw alignment files were cleaned using samtools v.1.2 (Li *et al*. 2009) and picard tools v.1.92 (http://picard.sourceforge.net/). PCR duplicates were removed, and only uniquely aligned reads in proper pairs were kept. This will remove most of the reads mapped to repetitive sequences, such as transposable elements, while retaining reads mapping to sequences that have been duplicated after the split of *Alloteropsis* and *Setaria*. The cleaned alignments were used to call single nucleotide polymorphic variants (SNPs) with samtools v. 0.1.19 using the mpileup function followed by the vcfutil.pl script with default setting supplied with the program. The South African C_4_ individual ‘RSA3’ was excluded during SNP calling to avoid any bias that might result from the presence of more than two alleles in this polyploid (see Lundgren *et al*. 2015 and Table S1, Supporting information). Genotypes of each accession, including ‘RSA3’, at all called SNP positions were extracted from the alignments using the mpileup function in samtools v.0.1.19, supplying the program with the positions of the called SNPs, and in‐house developed scripts for further processing (Appendix S1, Supporting information). The low‐coverage data caused genotype probabilities to be low, which precluded effective filtering based on these probabilities. Therefore, fixed genotype calls were used. To evaluate the proportion of SNPs corresponding to exon sequences, annotations were extracted for the 25 727 coding regions of the *Setaria* genome with homologs in maize and rice genomes (from now on referred to as SZR homologs). The positions of the raw SNPs were intersected with the SZR homolog annotations in bedtools v.2.19.1 using default settings (Quinlan & Hall 2010).

SNPs with coverage above 2.5 times the genomewide coverage (Table S2, Supporting information) were converted to unknown genotype calls. Furthermore, genotypes with more than two allele calls were also converted to missing data, and finally, positions with more than 50% missing data/unknown genotypes were discarded. The remaining 170 629 positions were used to infer a phylogenetic tree, using PhyML (Guindon *et al*. 2010) and a GTR substitution model (the best fit model as determined by hierarchical likelihood ratio tests), after coding heterozygous sites with IUPAC codes. Support was evaluated with 100‐bootstrap pseudoreplicates. The low‐coverage data likely cause some alleles to be missed, leading to an overestimate of homozygosity. However, no bias is expected in the missing allele, so that the low coverage is unlikely to lead to spurious groupings.

To test for a bias due to uneven coverage across samples (Table S2, Supporting information), we repeated the phylogenetic analysis on a resampled alignment, where all samples have the same number of bases mapped to the *Setaria* genome. Reads were randomly sampled without replacement from the filtered alignment files until the number of bases across the sampled reads equalled that of the sample with the lowest coverage (Appendix S2, Supporting information). These reanalyses were first conducted with all samples, which resulted in a low number of positions constrained to the samples with the lowest coverage. While analyses on the resampled data set were consistent with the whole‐data set analyses, the limited number of characters resulted in reduced support. We consequently repeated the resampling allowing for the full alignment of the two *A. semialata* samples with the lowest coverage and alignment success (‘AUS1’ and ‘RSA2’) to be retained at a slightly lower coverage than the rest of the samples. SNPs were called as outlined above, which allowed for the retention of 22 821 SNPs.

### Genetic structure and test for secondary gene flow

Preliminary cluster analyses with a focus on *A. semialata* showed that a more stringent filtering of the SNPs improved convergence of the analyses. Only positions with <10% missing data (2607 SNPs) within this species were consequently kept for analyses of its population structure, using the structure software (Pritchard *et al*. 2000). Ten independent analyses were run for each number of population components (*K*) from one to ten, under the admixture model. The adequate run length and burn‐in periods were determined through preliminary analyses, which indicated that a burn‐in period of 300 000 generations followed by 200 000 iterations provided stable estimates for all *K* values. The optimal *K* values were determined using the method of Evanno *et al*. (2005), as implemented in structureharvester (Earl & vonHoldt 2012). The results of the ten runs for each *K* were summarized using clumpp v. 1.1.2 (Jakobsson & Rosenberg 2007) and graphically displayed using distruct v. 1.1 (Rosenberg 2004). These analyses were repeated without the polyploid individual ‘RSA3’, which led to the same cluster assignments, showing that differences in ploidy levels do not affect the conclusions. Finally, the cluster analyses were repeated on alignments based on the reads subsampled to similar coverage in all sampled, allowing for 25% missing data per site (retention of 681 SNPs).

Different relationships among fractions of the nuclear genome can result from reticulated evolution or incomplete lineage sorting (Green *et al*. 2010; Durand *et al*. 2011). To distinguish these two possibilities, the ABBA–BABA method, which relies on the *D* statistic to test for asymmetry in the frequencies of incongruent phylogenetic groupings (Green *et al*. 2010; Durand *et al*. 2011), was used to test for secondary gene flow on a genomewide level in cases suggested by phylogenetic and clustering analyses (see Results). The low coverage likely leads to an overestimate of homozygous sites, but no bias is expected towards ABBA or BABA sites, leaving estimations of distorted gene flow unaffected. For each test, a four‐taxon phylogeny was selected, consisting of an outgroup and three tips among which secondary gene flow is suspected. Reads mapping to the 170 629 SNPs were recovered from the filtered alignment files using bedtools v.2.19.1 by intersecting the alignment files with positional information of the SNPs using default settings. The recovered reads were evaluated using the ‐doAbbababa option in the angsd program version 0.911 (Korneliussen *et al*. 2014). A jackknifed estimate of the *D* statistic and the corresponding *Z*‐value were obtained by the jackknif.R script supplied with the angsd program.

### Assembly and analyses of selected genes

Two different groups of closely related genes were selected for detailed analyses. The genes selected were two C_4_‐related protein‐coding genes, phosphoenolpyruvate carboxylase (*ppc* genes) and phosphoenolpyruvate carboxykinase (*pck* genes), that include some copies acquired by *Alloteropsis* from distantly related species via lateral gene transfer, while other copies were vertically inherited following the species tree (Christin *et al*. 2012). Previous conclusions regarding the distribution of these genes among accessions of *Alloteropsis* were based on PCR and Sanger sequencing, which can be biased due to the possibility of primer binding mismatches. The presence/absence of the laterally acquired *ppc* and *pck* genes and their vertically inherited homologs across the accessions were therefore re‐evaluated here using the genome‐skimming data, as well as new PCR and Sanger sequencing with primer verified against the new genomic data. Using molecular dating, the divergence times of the laterally acquired genes were compared to those of vertically inherited homologs belonging to the same set of accessions.

Reads were first mapped on gene segments of the *ppc* and *pck* genes from different accessions of *Alloteropsis* (grass co‐orthogols *ppc‐1P3* and *pck‐1P1*; Christin *et al*. 2012, 2015). These segments have been previously sequenced and analysed in a number of other C_3_ and C_4_ grasses (Christin *et al*. 2012). The availability of this rich reference data set allows mapping to closely related accessions of *Alloteropsis*, which improves the alignment success compared to the whole‐genome approach described above, and increases the confidence in the assignment. The gene segments cover exons 8–10 for *ppc* and exons 3–10 for *pck*, including introns, and represent approximately 46% (1492 bp) and 63% (1487 bp), respectively, of the full‐length coding sequences. In‐house Perl scripts (Appendix S3, Supporting information) were used to unambiguously assign reads to genes of these data sets, following the phylogenetic annotation method of Christin *et al*. (2015). In summary, this approach consists of: (i) building a reference data set of sequences with known identity for closely related gene lineages, (ii) using blast searches to identify all sequences homologous to any of these reference sequences in the query data set (the filtered reads in this case), (iii) independently aligning each homologous sequence to the reference data set and inferring a phylogenetic tree and (iv) establishing the identity of each of the query sequences based on the phylogenetic group in which it is nested. Assignment of reads to the gene lineages was verified by visual inspection of the phylogenetic trees and the alignments. Subsequently, all reads assigned to each of the vertically inherited or laterally acquired gene lineages were retrieved, and aligned to PCR‐isolated sequences (see Results) using geneious v. 6.8 (Kearse *et al*. 2012). The reads were then assembled into gene models, comprising introns and exons, for the studied segments. Multiple gene models were assembled for a single individual when the existence of distinct alleles was supported by at least two different polymorphic sites, each with at least two independent reads. Paired‐end reads were then merged into contigs if they shared the polymorphisms. Reads that did not overlap the polymorphic sites were merged with all alleles, replacing additional polymorphisms with IUPAC ambiguity codes.

To check whether partial pseudogenes that do not include the studied segments exist in some genomes, the presence of laterally acquired *ppc* genes was also tested using only coding sequences corresponding to exons 1–7, which were retrieved from a transcriptome study of *A. semialata* (Christin *et al*. 2012). This transcriptome was generated for a South African C_4_ polyploid with two laterally acquired *ppc* genes, but the vertically inherited versions of *ppc* and *pck* were not available in this transcriptome, preventing phylogenetic analyses. Blastn searches were used to identify reads mapping to one of the two laterally acquired *ppc* genes on at least 50 bp with at least 99% of identity. Finally, the presence/absence of the different *pck* and *ppc* copies was further confirmed via PCR and Sanger sequencing using primers specific to the different gene copies (Table S3, Supporting information; Christin *et al*. 2012). PCR, purification and sequencing were conducted as described in Lundgren *et al*. (2015), except for changes of the annealing temperature and/or extension time (Table S3, Supporting information). These PCR were conducted only on samples for which good quality DNA was available. Indeed, DNA isolated from herbarium samples is highly degraded, precluding reliable PCR screening.

To verify the gene models assembled from genome skimming for *ppc* and *pck*, the PCR amplified and Sanger sequenced fragments of the vertically inherited and laterally acquired genes were added to the genes assembled from short‐read data. The data sets were aligned using muscle v3.8.31 (Edgar 2004) with default parameters, and the alignments were manually refined. Maximum‐likelihood phylogenetic trees were inferred using PhyML, under a GTR+G model, and with 100‐bootstrap pseudoreplicates. Molecular dating was performed on the same alignments using beast as described above for chloroplast markers, but with a coalescent prior. The Andropogoneae/Paspaleae group (represented by *Sorghum*,* Paspalum* and one of the laterally acquired *ppc*) was selected as the outgroup, and the root of the tree was calibrated with a normal distribution with a mean of 31 Ma, and a standard deviation of 0.0001, as previously estimated for this node (Christin *et al*. 2014).

## Results

### Read alignment and SNP calling

The number of filtered paired‐end reads varied across samples, for a genomewide coverage ranging from 0.70 to 4.52 (Table S2, Supporting information). The proportion of filtered paired‐end reads that aligned to the *Setaria* genome varied between 4.04% and 10.23%, and between 1.22% and 2.94% aligned to the coding regions (Table S2, Supporting information). While the mapping was performed across the whole genome of *Setaria* (excluding the organelle genomes), divergence of noncoding sequences means that high mapping success is expected to be concentrated mostly onto coding sequences. About 9% of the *Setaria* genome corresponds to exons (Bennetzen *et al*. 2012). Assuming that the total length of exons is similar in the two species, the larger genome of *Alloteropsis* means that this proportion should be about 4.5%, so that approximately half of the reads corresponding to nuclear exons were mapped. The rest of the reads that belong to exons probably correspond to gene sections that are too divergent between the two species to successfully map.

Only uniquely aligned reads were used to call SNPs, which inherently excludes common repetitive regions such as transposons. However, 1111 raw SNPs had a higher than expected coverage (>5×) across at least 50% of the samples. The positions of 91% (1007) of these high‐coverage SNPs fell outside of the SZR homolog regions, and the rest were concentrated to only 14 SZR homologs. We therefore hypothesize that these high‐coverage SNPs stem from genetic regions (mostly noncoding) that have been duplicated after the split between *Alloteropsis* and *Setaria* and they were subsequently removed from the analyses.

A total of 170 629 SNPs with <50% missing data across the 21 accessions were finally selected for downstream analyses. These sites are spread across all chromosomes (Fig. S1, Supporting information) and 96% of them fall within one of 9948 SZR homologs. The 2607 SNPs used for the cluster analysis (<90% missing data across the *Alloteropsis semialata* samples) were equally well spread across the genome (Fig. S1, Supporting information) and 97% fall within one of 848 SZR homologs. Most of the variation in missing data across samples (Table S2, Supporting information) is likely explained by differences in coverage, although the presence/absence of genes within each accession might also influence the individual mapping success.

Overall, our analyses show that our pipeline, despite a low overall coverage and low alignment success due to the large divergence time between *Alloteropsis* and *Setaria*, captures variation in almost 10 000 genes spread across the genomes of grasses.

### Phylogenetic trees

**Figure 2:**
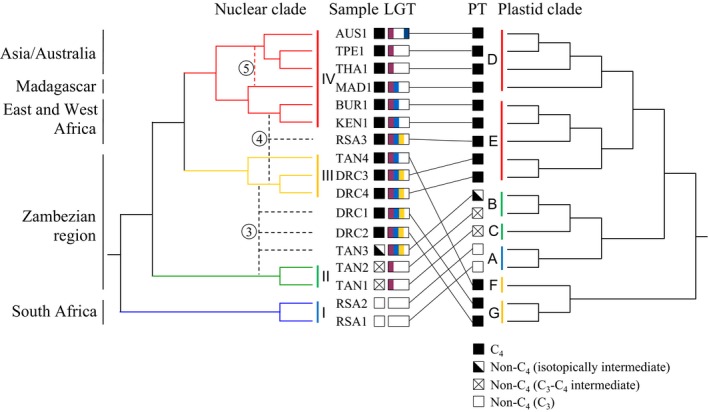
Comparison of nuclear (on the left) and plastid (on the right) topologies (without branch lengths). The putative origin of individuals with mixed genetic back ground was added using dashed lines. Branches of the nuclear tree are coloured according to clustering analyses (Fig. 3). Photosynthetic types (PT) and presence of laterally acquired genes (LGT) are indicated by symbols at the tips; purple bar = presence of *pck‐1P1_LGT:C*, blue bar = *ppc‐1P1_LGT:M*, orange bar = *ppc‐1P1_LGT:C*, dark blue bar = *ppc‐1P1_LGT:A*. Geographic origin is indicated on the left. Secondary gene flow is numbered as in Fig. 6; (3) hybridization between non‐C_4_ and C_4_ populations within the Zambezian region, (4) allopolyploidy between C_4_ populations in Africa (‘RSA3’), (5) pollen‐mediated gene flow from mainland Africa to Madagascar.

The plastid phylogeny identified two C_4_ individuals from DRC with haplotypes that form a new C_4_ plastid lineage based on divergence times (i.e. lineage G, sister to lineage F; Figs 2 and S2, Supporting information). Relationships based on markers sampled across the nuclear genome confirm the monophyly of *A. semialata* and its sister‐group relationship to *Alloteropsis angusta*, but present multiple incongruences with the chloroplast tree within *A. semialata* (Figs 2 and S3, Supporting information). In this genomewide tree, the first divergence leads to a group composed of the non‐C_4_ accessions of *A. semialata* from South Africa without any known C_4_ cycle (Clade I; Fig 2 and S3, Supporting information), and the second divergence leads to a group comprising the non‐C_4_ accessions from the Zambezian region that use a weak C_4_ cycle (Clade II; Fig 2 and S3, Supporting information; C_3_‐C_4_ intermediates sensu Lundgren *et al*. 2016). The isotopically intermediate accession ‘TAN3’ is then sister to all C_4_ accessions (Figs 2 and S3, Supporting information). The two C_4_ accessions bearing the plastid lineage G form a paraphyletic clade, while the other C_4_ accessions from the Zambezian region (‘TAN4’, ‘DRC3’ and ‘DRC4’) are grouped in a strongly supported clade (Clade III; Figs 2 and S3, Supporting information). The South African polyploid individual ‘RSA3’ is sister to the C_4_ individuals sampled outside of the Zambezian region, and the rest of the C_4_ accessions form the strongly supported clade IV, with two subclades corresponding to Africa plus Madagascar and Asia plus Australia (Figs 2 and S3, Supporting information). The nuclear phylogeny based on the resampled data set is mostly identical to the one based on the whole data set (Figs S3 and S4, Supporting information).

### Genetic structure and secondary gene flow within *Alloteropsis semialata*

**Figure 3:**
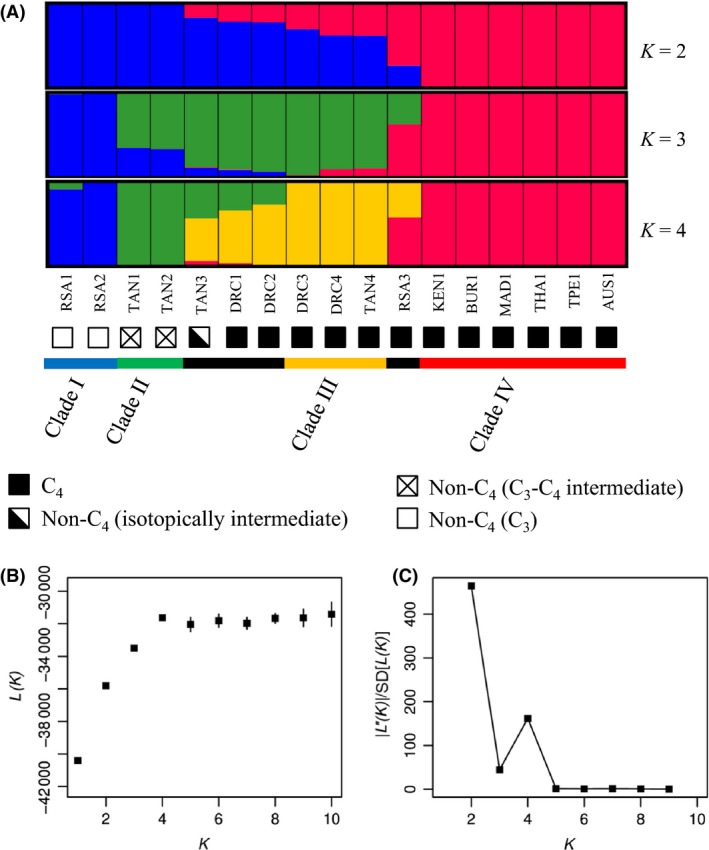
Assignment of *Alloteropsis semialata* individuals to genetic clusters. (A) Assignment of each individual to the different clusters (*K* 2–4). The photosynthetic type is indicated by symbols next to the names, as in Fig. 2. (B) Mean likelihood (±SD) over 10 runs for each *K* value (1–10), and (C) |L′′(*K*)|/SD (fit improvement) as calculated according to Evanno *et al*. (2005).

Based on the whole‐genome clustering analysis, four clusters explain the data set best, and adding groups does not improve the likelihood (Fig. 3B). However, the method of Evanno *et al*. (2005) indicates that the maximum fit improvement is at two clusters, with four clusters representing the second maximum fit improvement (Fig. 3C). With four clusters, the main clades from the genome wide phylogeny are recovered (Figs 2 and 3A). This genetic structure matches the photosynthetic types rather than the geographic origin, with the non‐C_4_ clades I and II and the C_4_ clades III and IV each forming distinct homogenous groups (Fig. 3A). The three Zambezian individuals that formed a paraphyletic clade in the nuclear phylogeny (‘TAN3’, ‘DRC1’ and ‘DRC2’) are partially assigned to two Zambezian groups, the non‐C_4_ clade II and the C_4_ clade III (Figs 2 and 3A). Finally, the polyploid individual from South Africa, ‘RSA3’, is partially assigned to the two C_4_ clades III and IV (Fig. 3A). The cluster results based on the resampled data set are less stable due to a lower number of sites, but the assignments are similar (Figs 3 and S5, Supporting information).

Heterozygosity was estimated for each sample based on the 22 821 SNPs from the resampled data set with similar coverage across samples. While these estimates are based only on sites variable within *Alloteropsis* and should consequently not be interpreted as genomewide heterozygosity, it is possible to compare the estimates between samples. The individuals assigned to multiple clusters had the highest percentage of heterozygous SNPs (Fig. S6, Supporting information), which confirms their genetic diversity.

**Figure 4:**
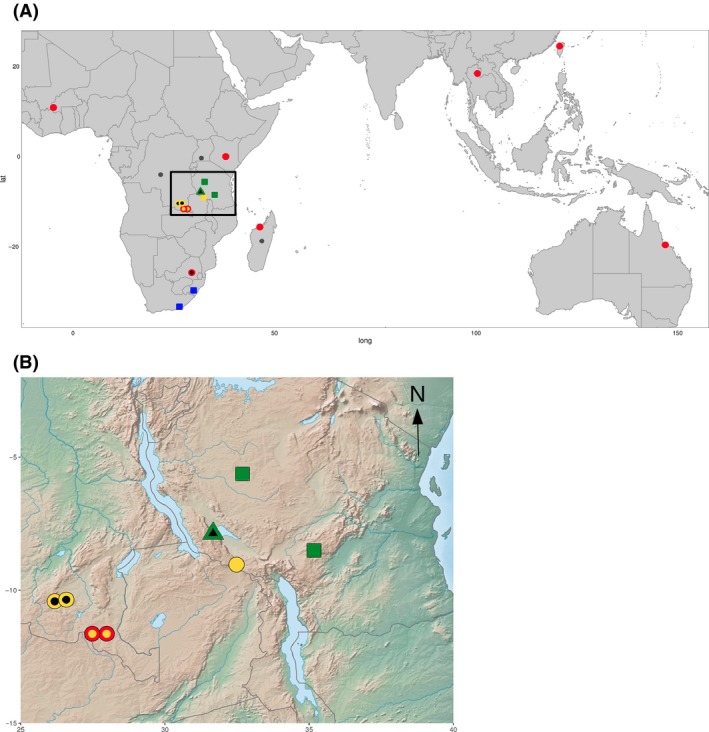
Geographic distribution of *Alloteropsis semialata* genetic lineages. (A) World distribution, highlighting the Zambezian region with a rectangle and (B) details of the Zambezian region. For each point, the colour of the outline indicates the plastid lineage (blue = clade A; green = clade BC; yellow = clade FG; red = clade DE), while the colour of the background represents the nuclear lineage (blue = clade I; green = clade II; yellow = clade III; red = clade IV; black = mixed genetic background; grey = congeners). Finally, the shape of the point indicates the photosynthetic type, as determined by carbon isotopes (square = non‐C_4_; circle = C_4_; triangle = isotopically intermediate).

Together, our intraspecific genetic analyses reveal the existence of distinct gene pools despite overlapping distributions (Figs 3A and 4), but also suggest that genetic exchanges have happened among groups. The incongruences between the phylogenetic structures of the chloroplast and nuclear genomes, together with the assignment of some individuals to multiple genetic clusters, suggest that the three Zambezian individuals ‘TAN3’, ‘DRC2’ and ‘DRC1’ have ancestors from distinct genetic groups, in this case the nuclear clades II and III. ABBA–BABA tests were therefore conducted to test this hypothesis, using *A. angusta* (individual Ang2) as the outgroup. The individual ‘TAN4’ was selected as the representative of clade III because it is geographically more distant and distinct on all genetic markers (Figs 4, S2 and S3, Supporting information). Significant indications (*P* < 0.05 after correction for multiple testing) of gene flow from the non‐C_4_ clade II (‘TAN2’ and ‘TAN1’) into the populations assigned to multiple clusters (‘TAN3’, ‘DRC2’ and ‘DRC1’) were found (Table 1). In contrast, there is no evidence of a significant secondary contribution of clade II into individuals of clade III (‘DRC3’ or ‘DRC4’; Table 1). However, in one case, a slight excess of BABA sited was detected, which was not significant after correction for multiple testing (Table 1). This would suggest some genetic contribution from one non‐C_4_ population of clade II (‘TAN1’) into the C_4_ population represented by ‘TAN4’ (Table 1).

**Table 1:** Results of ABBA–BABA tests

Outgroup^a^	P3^a^	P2^a^	P1^a^	# ABBA sites	# BABA sites	*D* b	*Z*	*P*‐value^c^	Conclusion
*Alloteropsis angusta*	TAN2	TAN3	TAN4	2630	1805	0.186	8.279	<0.0001	TAN2 closer to TAN3 than to TAN4
*A. angusta*	TAN1	TAN3	TAN4	2630	1750	0.201	8.757	<0.0001	TAN1 closer to TAN3 than to TAN4
*A. angusta*	TAN2	DRC2	TAN4	2037	1546	0.137	5.939	<0.0001	TAN2 closer to DRC2 than to TAN4
*A. angusta*	TAN1	DRC2	TAN4	1960	1570	0.110	4.724	<0.0001	TAN1 closer to DRC2 than to TAN4
*A. angusta*	TAN2	DRC1	TAN4	2240	1752	0.122	5.463	<0.0001	TAN2 closer to DRC1 than to TAN4
*A. angusta*	TAN1	DRC1	TAN4	2194	1749	0.113	5.692	<0.0001	TAN1 closer to DRC1 than to TAN4
*A. angusta*	TAN2	DRC4	TAN4	1177	1164	0.006	0.223	0.824	TAN2 equally close to DRC4 and TAN4/correct phylogeny
*A. angusta*	TAN1	DRC4	TAN4	1075	1123	−0.022	−0.866	0.386	TAN1 equally close to DRC4 and TAN4/correct phylogeny
*A. angusta*	TAN2	DRC3	TAN4	1372	1451	−0.028	−1.080	0.280	TAN2 equally closer to DRC3 and TAN4/correct phylogeny
*A. angusta*	TAN1	DRC3	TAN4	1248	1431	−0.068	−2.885	0.004^d^	TAN1 might be closer to TAN4 than to DRC3
TAN4	TPE1	MAD1	BUR1	1314	1129	0.076	2.603	0.009^d^	TPE1 might be closer to MAD1 than to BUR1

a (Outgroup,(P3,(P2,P1))).

b *D* statistic: (ABBA‐BABA)/(ABBA+BABA).

c *P*‐value for *Z* score as calculated by jackknife for whether *D* differs significantly from zero.

d Nonsignificant after Bonferroni correction for multiple testing.

Within clade IV, the C_4_ individual from Madagascar (‘MAD1’) was grouped with Asian C_4_ accessions on plastid genomes but grouped with the African C_4_ accessions based on markers from across the nuclear genome (Figs S2 and S3, Supporting information). An ABBA–BABA test was conducted to test the hypothesis of secondary gene flow after the split of the African and Asian C_4_ accessions. The accession ‘TAN4’ was used as the outgroup, being sister to all accessions from clade IV. The Taiwan accession (‘TPE1’) was selected as the Asian sample, while the Burkinabe accession (‘BUR1’) represented Africa. Overall, more ABBA than BABA sites were detected (Table 1), indicating that the Asian accession was closer to the accession from Madagascar (‘MAD1’) than to the accession from mainland Africa, but the *D* statistic for this test was not significant after correcting for multiple testing (Table 1). Plastid markers, which represent seed dispersal, group the Madagascan accessions with Asian individuals. Therefore, a possible scenario involves an initial seed dispersal from Africa to Madagascar and then from Madagascar to Asia, with subsequent pollen flow between Africa and Madagascar.

### Assembly and analyses of selected genes

The presence/absence of *ppc* and *pck* genes was established by mapping reads directly onto reference sequences from *Alloteropsis*. The distribution of the genes was also confirmed by PCR followed by Sanger sequencing (Fig. S7, Supporting information).

**Figure 5:**
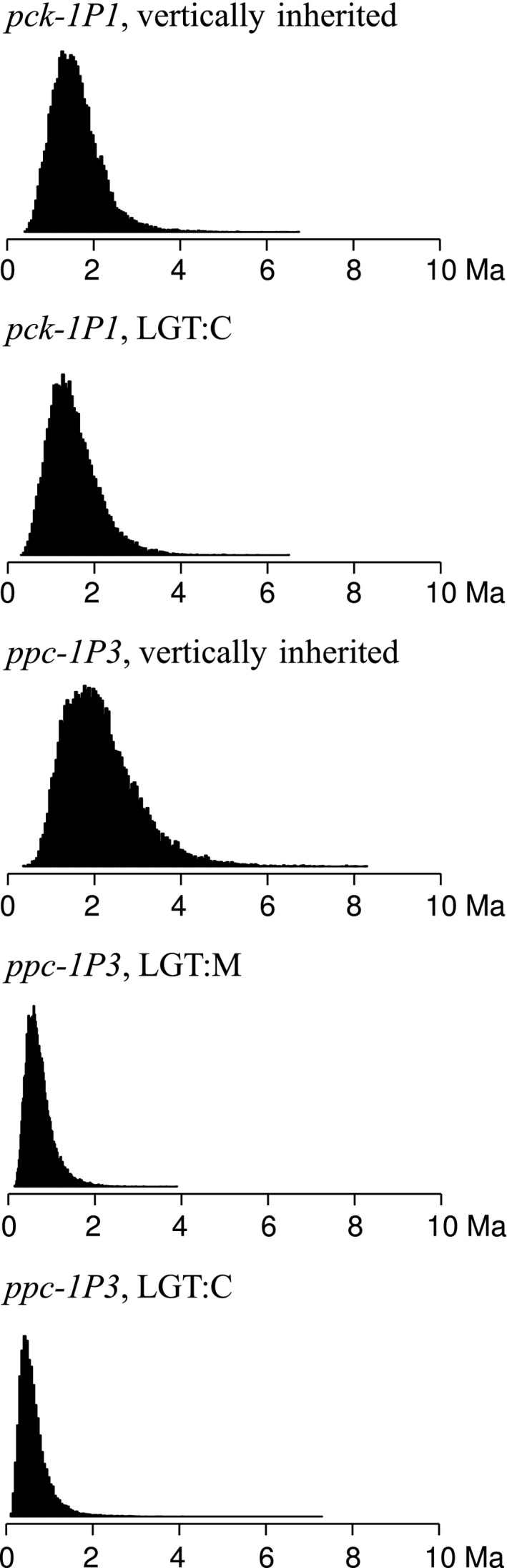
Divergence times of C_4_ accessions of *Alloteropsis semialata* based on vertically inherited and laterally acquired genes. For five *ppc* and *pck* genes, the posterior distribution of times to the last common ancestor of the C_4_ *A. semialata* is shown, in million years (Ma).

Together, the results confirmed previous phylogenetic analyses (Christin *et al*. 2012), but with a significant increase of the sample size. Reads assigned to the *pck* gene copy laterally acquired from members of the *Cenchrus* genus (*pck‐1P1_LGT:C*) were detected in the two *A. angusta* accessions and all *A. semialata* accessions, except the two non‐C_4_ accessions from South Africa (Table 2; Figs S7 and S8, Supporting information). The sequences assembled for the laterally acquired *pck* gene were highly similar between the different accessions, leading to a poorly resolved phylogeny (Fig. S8, Supporting information). By contrast, the sequences assembled for the vertically inherited *pck* gene were variable among accessions, and the nuclear clades I and II were recovered in their phylogeny, while clades III and IV were not differentiated (Fig. S8, Supporting information). Interestingly, one accession with mixed genetic backgrounds (‘DRC1’) has two divergent alleles, one of which groups with clade II and the other with clade III/IV (Fig. S8, Supporting information). Dating analyses indicate that the divergence of *A. angusta* and *A. semialata* is more recent for the laterally acquired *pck* than for the vertically inherited copy (Fig. S9, Supporting information). However, the divergence of C_4_ accessions of *A. semialata* is estimated at a similar time based on the vertically inherited and laterally acquired *pck* (Fig. 5).

**Table 2:** Number of read assigned to each of the laterally acquired *pck* and *ppc* genes

Species	Accession	Phylogenetic group (plastid; nuclear)	*ppc‐1P3_LGT:A* a	*ppc‐1P3_LGT:M* b	*ppc‐1P3_LGT:C* c	*pck‐1P1_LGT:C* c
*Alloteropsis cimicina*	Cim1	–	0	149^d^	0	0
*Alloteropsis paniculata*	Pan1	–	0	37^d^	0	0
*Alloteropsis angusta*	Ang2	–	0	0	0	78
	Ang1	–	0	0	0	49
*Alloteropsis semialata*	RSA1	A; I	0	0	0	0
	RSA2	A; I	0	0	0	0
	TAN1	C; II	0	0	0	57
	TAN2	B; II	0	0	0	73
	TAN3	B; mixed	0	54^d^	0^e^	216^d^
	DRC1	G; mixed	0	57^d^	56	183^d^
	DRC2	G; mixed	0	29	50^d^	95^d^
	DRC3	E; III	0	6	25	135^d^
	DRC4	E; III	0	10	12	88^d^
	TAN4	F; III	0	76	0	83
	RSA3	E; IV	0	55^d^	63	113
	KEN1	E; IV	0	36	0	85
	BUR1	E; IV	0	26	0	130
	MAD1	D; IV	0	46	0	101
	THA1	D; IV	0	0	0	123
	TPE1	D; IV	0	0	0	118
	AUS1	D; IV	55	0	0	110

a Laterally acquired from Andropogoneae.

b Laterally acquired from Melinidinae.

c Laterally acquired from Cenchrinae.

d Assembly of more than one allele.

e Note that seven reads were retrieved for exons 1–7, which indicates that this gene is truncated in the genome of this accession.

The vertically inherited *ppc* was recovered from all samples, and the assembled gene models were variable enough to partially resolve the phylogeny, with well‐supported clades corresponding to the different species (Fig. S10, Supporting information). Although support was limited within *A. semialata*, the non‐C_4_ clades I and II (including sequences from individuals assigned to multiple clades) were sister to a clade composed of the C_4_ accessions from clade IV nested within those of clade III (Fig. S10, Supporting information). The divergence of vertically inherited *ppc* from C_4_ accessions (excluding those partially assigned to clusters other than III and IV) matches the divergence of the vertically inherited *pck* for the same accessions (Fig. 5).

The *ppc* gene laterally acquired from Andropogoneae (*ppc‐1P3_LGT:A*) was only detected in the Australian C_4_ accession (‘AUS1’; Table 2, Figs S7 and S10, Supporting information). An almost complete sequence for the studied segment was assembled, which was identical to those isolated by PCR.

The *ppc* gene laterally acquired from the *Setaria palmifolia* complex (*ppc‐1P3_LGT:C*) was detected in the C_4_ accessions from South Africa (‘RSA3’) and the DRC (Table 2, Figs S7 and S10, Supporting information). Although no reads matching exons 8–10 of *ppc‐1P3_LGT:C* were detected in the accession ‘TAN3’, a total of seven reads from this individual matched exons 1–7. This suggests that the gene is truncated and probably exists as a pseudogene in this individual. The *ppc‐1P3_LGT:C* sequences were largely conserved, although distinct alleles were assembled in one of the accessions with mixed genetic background (‘DRC2’; Fig. S10, Supporting information). The divergence of *ppc‐1P3_LGT:C* genes belonging to different C_4_ accessions was more recent than for the vertically inherited *ppc* and *pck* of the same accessions (Fig. 5).

The *ppc* gene acquired from Melinidinae (*ppc‐1P3_LGT:M*) was identified in nine C_4_ accessions of *A. semialata*, the isotopically intermediate *A. semialata*, and the two congeners *Alloteropsis cimicina* and *Alloteropsis paniculata* (Table 2, Figs S7 and S10, Supporting information). Highly divergent alleles of the *ppc‐1P3_LGT:M* gene were inferred for *A. cimicina* and *A. paniculata* (Fig. S10, Supporting information). However, the sequences of *ppc‐1P3_LGT:M* from *A. semialata* were very similar to each other, and nested within the alleles from *A. cimicina*/*paniculata* (Fig. S10, Supporting information). The split of *A. semialata* and *A. cimicina* is more recent for *ppc‐1P3_LGT:M* than for the vertically inherited *ppc* and *pck* (Fig. S9, Supporting information). In addition, the divergence of C_4_ accessions of *A. semialata* based on this *ppc‐1P3_LGT:M* gene occurred more recently than the divergence based on the vertically inherited *ppc* and *pck* (Fig. 5).

## Discussion

### Divergence of photosynthetic types in isolation followed by secondary gene flow

**Figure 6:**
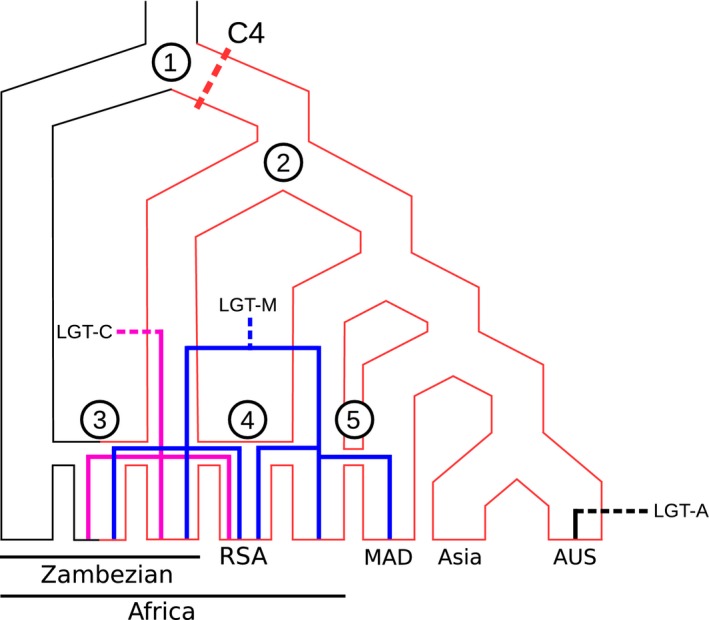
Inferred history of divergence, secondary exchanges and spread of laterally acquired *ppc* genes in *A. semialata*. A summary phylogeny is shown for the C_4_ and non‐C_4_ accessions of *A. semialata*, excluding the non‐C_4_ individuals from South Africa. The C_4_ phenotype is represented with red outlines. (1) The divergence of photosynthetic types is inferred in the Zambezian region (dashed red line indicates C_4_ emergence). (2) A C_4_ lineage migrated outside of the Zambezian region. (3) Hybridization occurred between non‐C_4_ and C_4_ populations within the Zambezian region. (4) The C_4_ polyploids of South Africa (RSA) resulted from segmental allopolyploidy. (5) Pollen‐mediated gene flow occurred from mainland Africa to Madagascar. The lateral acquisition of three *ppc* genes is indicated with dashed lines, and their subsequent spread is indicated with solid lines. Geographic regions are indicated at the bottom.

Overall, our genomewide analyses reveal a strong genetic structure, which matches photosynthetic types better than geographic origins, although both play a role. All C_4_ individuals form a monophyletic group based on genomewide markers, which is sister to a clade composed of non‐C_4_ accessions from the Zambezian region with a weak C_4_ cycle (clade II; Figs 2 and 4), and together, these two groups are sister to the non‐C_4_ accessions from South Africa that lack a C_4_ cycle (clade I; Figs 2 and 4). The C_4_ clade contains two clearly distinct subgroups, one from the Zambezian region (clade III; Figs 2 and 4) and the other one encompassing all C_4_ accessions sampled outside this region, from Western Africa to Australia (clade IV; Figs 2 and 4). The Zambezian region encompasses more genetic diversity than the rest of the species’ range, including a total of five plastid lineages, four of which are endemic (clades B, C, F and G; Figs 2 and S2, Supporting information). This finding further supports this region as the centre of origin for *Alloteropsis semialata* (Lundgren *et al*. 2015). Based on both plastid and nuclear genomes, the divergence of photosynthetic types likely also happened within this region (Fig. 6). Both C_4_ and non‐C_4_ populations in the Zambezian region are associated with Miombo woodlands. Periodic cycles of contraction and expansion of these wooded savannas during recent geological times might have isolated populations of *A. semialata* in this geologically and topographically complex region (Cohen *et al*. 2007; Beuning *et al*. 2011). The ancestral photosynthetic state is likely non‐C_4_ and mutations altering the leaf anatomy and upregulation of enzymes already present in the non‐C_4_, ancestors likely led to the emergence of a constitutive C_4_ cycle in some isolated populations (Mallmann *et al*. 2014; Bräutigam & Gowik 2016). One of the lineages descending from the initial C_4_ pool, corresponding to clade IV, later left the Miombo of the Zambezian region and rapidly spread across Africa and all the way to Asia and Australia (Figs 4 and 6). This biogeographical history therefore points to the initial emergence of the C_4_ physiology in *A. semialata* within the Zambezian region, with subsequent isolation of the C_4_ descendants (Fig. 6).

The lack of association between chloroplast and nuclear groups (Figs 2, S2 and S3, Supporting information) in the Zambezian region suggests ancient, but recurrent, secondary gene flow followed by homogenization of the local gene pools. In addition, the presence of three individuals with mixed nuclear backgrounds indicates relatively recent gene flow between previously isolated groups. The maximum expansion of the Miombo woodlands during interglacial periods, as presently occurs, would likely favour seed dispersal over a larger area, leading to secondary contacts (Vincens 1989; Cohen *et al*. 2007; Beuning *et al*. 2011), a process frequently reported in Europe (reviewed in, e.g. Hewitt 2000; Schmitt 2007). We propose that this expansion allowed genetic exchanges between previously isolated lineages, some of which had made the transition to a full C_4_ physiology during the previous isolation. No evidence of gene flow between C_4_ and non‐C_4_ individuals was found outside of the Zambezian region, and crosses might be prevented in South Africa, the other region where C_4_ and non‐C_4_ individuals overlap, by differences in ploidy levels (Fig. 4; Liebenberg & Fossey 2001). However, our analyses suggest that allopolyploidy contributed to the mixing of nuclear groups III and IV in Southern Africa (Fig. 6). In addition, while the recent divergence decreases statistical confidence, we found suggestions for secondary gene flow between different subgroups of the C_4_ clade IV in Madagascar (Fig. 6).

Repeated isolation followed by recurrent, but rare secondary gene flow has created a dynamic population structure whereby adaptive mutations, such as those for the C_4_ trait, can appear and sweep to fixation in isolation and later come together through admixing in times of contact. While mutations for increasing the expression of the C_4_‐related genes and altering the leaf anatomy are unknown, genes for two key C_4_ enzymes were laterally acquired by *A. semialata* (Christin *et al*. 2012). These lateral gene transfers likely took place in *A. semialata* plants that already used C_4_ photosynthesis, and once acquired, these genes presumably replaced the function of the vertically inherited gene copies that were overexpressed but not biochemically optimized (Christin *et al*. 2012). The biogeographic history inferred here for the nuclear genome allows us to estimate the region where these lateral gene transfers likely occurred and track the subsequent spread of these genes among different gene pools.

### Spread of C_4_‐adaptive mutations among gene pools

Our analyses detected the laterally acquired *pck* gene in all *Alloteropsis angusta* and *A. semialata* individuals apart from two non‐C_4_ *A. semialata* South African accessions of *A. semialata* from South Africa, confirming previous PCR‐based approaches (Table 2; Christin *et al*. 2012). The divergence time is younger between the laterally acquired *pck* genes from *A. angusta* and *A. semialata* than between the vertically inherited genes of the same species (Figs 5 and S9, Supporting information). This suggests that the laterally acquired *pck* was passed between *A. angusta* and *A. semialata* through secondary gene flow.

The accessions from Taiwan and Thailand do not possess any laterally acquired *ppc* genes, yet carbon isotopes unambiguously indicate that they carry out C_4_ photosynthesis (Table 2; Lundgren *et al*. 2015). It is therefore likely that they overexpress their vertically inherited *ppc* and other genes required to generate a working C_4_ cycle in the absence of repeated rounds of fixation of adaptive amino acids, as observed in older C_4_ lineages (Christin *et al*. 2007; Besnard *et al*. 2009; Huang *et al*. in press).

Out of the three different *ppc* genes acquired via lateral gene transfers from distant C_4_ relatives (Table 2; Christin *et al*. 2012), only *ppc‐1P3_LGT:A* is restricted to one of the accessions sampled here (‘AUS1’). This gene was only found in Australia, and it is thus likely that it was recently acquired in this region (Fig. 6). The other two laterally acquired *ppc* genes are absent from some individuals, but spread across multiple populations of *A. semialata* that belong to different genomic clusters (Table 2). This pattern could result from the presence of the gene in the common ancestor and subsequent losses in some populations. However, this scenario is not supported by the lack of phylogenetic structure on the laterally acquired genes (Fig. S10, Supporting information) and the comparison of divergence times, which indicate that the divergence of variants of both *ppc‐1P3_LGT:M* and *ppc‐1P3_LGT:C* found in C_4_ accessions is more recent than the divergence of vertically inherited genes in the same accessions (Fig. 5).

The laterally acquired *ppc‐1P3_LGT:M* gene was identified in the C_4_ congeners *Alloteropsis cimicina* and *Alloteropsis paniculata*, as well as all C_4_ accessions of *A. semialata* from Africa and Madagascar (whether from clade III or IV; Table 2). However, this gene was absent from the Asian/Australian C_4_ accessions from clade IV and the African non‐C_4_ (clades I and II; Table 2). The divergence time between *ppc‐1P3_LGT:M* genes belonging to *A. cimicina* and *A. semialata* is younger than the divergence times for the vertically inherited genes from the same species (Fig. S9, Supporting information). In addition, the higher allelic diversity in *A. cimicina* compared to *A. semialata* suggests that the *ppc‐1P3_LGT:M* gene was first acquired by *A. cimicina* and then transferred to *A. semialata*, potentially via hybridization. This gene has subsequently likely spread across distinct genetic groups of *A. semialata* in Africa and Madagascar via secondary pollen flow (Fig. 6). The fixation of the *ppc‐1P3_LGT:M* gene within different populations would have been favoured by its improvement of the C_4_ cycle, a function for which it was already optimized after millions of years spent in another C_4_ lineage. Once this adaptive gene copy was acquired in a population, the vertically inherited *ppc* copy probably underwent pseudogenization as a result of relaxed selection. Indeed, the vertically inherited *ppc* genes bear frameshift mutations causing loss of function in two accessions with the laterally acquired *ppc‐1P3_LGT:M* (‘TAN4’ and ‘Cim1’), supporting the hypothesis that their function was taken over by the newly acquired gene, making them obsolete.

The last of the laterally acquired *ppc* genes, *ppc‐1P3_LGT:C*, was found in the South African C_4_ polyploid (‘RSA3’) as well as in four C_4_ and one isotopically intermediate individuals from the Zambezian region, two from clade III and three with genetic contributions from clades II and III (Table 2). This gene was laterally acquired from a species of the *Setaria palmifolia* complex (Christin *et al*. 2012), which co‐occurs with *A. semialata* in Zambezian Africa, where they grow metres apart, but not in South Africa (Clayton 1979). The transfer therefore likely occurred in the Zambezian region and later spread among the C_4_ populations in this region through secondary gene flow (Fig. 6). Once acquired the *ppc‐1P3_LGT:C* gene presumably took over the C_4_ function, which might have been fulfilled by the previously acquired *ppc‐1P3_LGT:M*. Indeed, *ppc‐1P3_LGT:M* is still expressed in the transcriptome of the South African C_4_ accession, but possesses internal stop codons that prevent proper translation (Christin *et al*. 2012). The newly acquired *ppc‐1P3_LGT:C* likely spread to the C_4_ populations from South Africa, through the putative segmental allopolyploidy event, providing a mechanism to propagate adaptive loci across genetic pools (Fig. 6). However, the Melinidinae *ppc‐1P3_LGT:M* discussed above was spread among diploid individuals from clades III and IV, showing that adaptive loci can be transmitted despite limited gene flow, without the need for polyploidization.

The laterally acquired genes, which can easily be tracked using genome scans, show that the distinct genetic pools in *A. semialata* constitute reservoirs of genes for the adaptation of other populations within the same species complex. The history of these markers proves that genes for a complex trait can evolve independently in isolated populations and later be combined via natural selection following gene flow. When high‐quality genome data accumulate for multiple accessions of *A. semialata*, such a scenario can be tested for vertically inherited genes, potentially explaining how novel adaptations can evolve in fragmented species complexes.

## Conclusions

In this study, we analysed genomic data from multiple accessions of the grass *Alloteropsis semialata* using low‐coverage whole‐genome sequencing. Using a biogeographic framework for different parts of the genome, we demonstrate that multiple genetic pools exist, which are generally associated with different photosynthetic types. These pools originated more than 2 million years ago in the Zambezian region and were kept relatively isolated, but with recurrent secondary gene flow, including between non‐C_4_ and C_4_ individuals. These genetic exchanges contributed to the spread of adaptive loci, as illustrated by key C_4_ genes acquired laterally in the Zambezian region and then rapidly passed to other African C_4_ accessions. This process likely gradually optimized the initial C_4_ pathway of some *A. semialata* populations through the assembly of different components. These genetic elements evolved in different parts of the species range, where limited gene flow might have facilitated local adaptation, but their subsequent combination likely improved the efficiency of the photosynthetic pathway of some accessions.

## Data accessibility

All raw reads are available in the short sequence archive under Accession no. SRP082653. In the NCBI nucleotide database, all newly assembled chloroplast genomes are available under Accession nos KX752083‐KX752090, *ppc* genes under Accession nos KX788072‐KX788087 and *pck* genes under Accession nos KX788088‐KX788109.

J.K.O, M.B., P.N., C.P.O. and P.A.C. designed the study. G.B., M.R.L., and M.S.V. provided samples. J.K.O., M.B., G.B., and H.H. generated the sequence data. M.R.L. generated the carbon isotope data. O.H. and I.J.L. generated the genome size data. J.K.O., M.B., L.T.D. and P.A.C. analyzed the data. J.K.O., M.B. and P.A.C. interpreted the results and wrote the paper, with the help of all co‐authors.

## Supporting information


**Figure S1** Distribution of the 171,908 called SNPs along the *Setaria italica* genome.
**Figure S2** Phylogenetic relationships based on complete chloroplast genomes from *Alloteropsis*.
**Figure S3** Phylogenetic relationships based on whole genome sequencing of *Alloteropsis* accessions.
**Figure S4** Phylogenetic relationships based on a sub‐set of the whole genome sequencing of*Alloteropsis* accessions.
**Figure S5** Assignment of *Alloteropsis semialata* individuals to genetic clusters based on a sub‐set ofthe aligned reads from the whole genome sequencing.
**Figure S6** Percentage of heterozygous sites for each accession.
**Figure S7** Results of PCR amplification of *ppc‐IP3* and *pck‐IP1* in *Alloteropsis*.
**Figure S8** Phylogeny of *pck‐1P1* in *Alloteropsis*.
**Figure S9** Divergence times for different nodes estimated from vertically‐inherited andlaterally‐acquired genes.
**Figure S10** Phylogeny of *ppc‐1P3* in *Alloteropsis*.
**Table S1** Sample and sequencing information.
**Table S2** Alignment statistics of the *Alloteropsis* genome‐skimming data to the *Setaria* reference genome.
**Table S3** Primer pairs for amplification of genes copies of phosphoenolpyruvate carboxylase (*ppc*) and phosphoenolpyruvate carboxykinase (*pck*).

**Appendix S1** Scripts used to genotype the samples and produce a phylip file and an input file for Structure.

**Appendix S2** Scripts used to resample a subset of reads.

**Appendix S3** Perl scripts used identify and retrieve reads corresponding to different *pck* and *ppc* gene lineages.
