# Supplementary material for: Genome biogeography reveals the intraspecific spread of adaptive mutations for a complex trait
Source: Mol Ecol. 2016 Nov 30;25(24):6107–23. doi: 10.1111/mec.13914 (PMC6849575; doi:10.1111/mec.13914)
Supplement: Supplementary file 1 — Figure S1 Distribution of the 171,908 called SNPs along the Setaria italica genome. Figure S2 Phylogenetic relationships based on complete chloroplast genomes from Alloteropsis. Figure S3 Phylogenetic relationships based on whole genome sequencing of Alloteropsis accessions. Figure S4 Phylogenetic relationships based on a sub‐set of the whole genome sequencing ofAlloteropsis accessions. Figure S5 Assignment of Alloteropsis semialata individuals to genetic clusters based on a sub‐set ofthe aligned reads from the whole genome sequencing. Figure S6 Percentage of heterozygous sites for each accession. Figure S7 Results of PCR amplification of ppc‐IP3 and pck‐IP1 in Alloteropsis. Figure S8 Phylogeny of pck‐1P1 in Alloteropsis. Figure S9 Divergence times for different nodes estimated from vertically‐inherited andlaterally‐acquired genes. Figure S10 Phylogeny of ppc‐1P3 in Alloteropsis. Table S1 Sample and sequencing information. Table S2 Alignment statistics of the Alloteropsis genome‐skimming data to the Setaria reference genome. Table S3 Primer pairs for amplification of genes copies of phosphoenolpyruvate carboxylase (ppc) and phosphoenolpyruvate carboxykinase (pck). [file MEC-25-6107-s001.pdf]

Supplementary material for:

Genome biogeography reveals the intraspecific spread of adaptive mutations for a complex trait

Authors: Jill K. Olofsson, Matheus Bianconi Guillaume Besnard, Luke T. Dunning, Marjorie R. Lundgren, Helene Holota, Maria S. Vorontsova, Oriane Hidalgo, Ilia Leitch, Patrik Nosil, Colin P. Osborne, Pascal-Antoine Christin

This Supplementary Material contains eleven figures, four tables, and three files:

Figure S1. Distribution of the 171,908 called SNPs along the *Setaria italica* genome.

Figure S2. Phylogenetic relationships based on complete chloroplast genomes from *Alloteropsis*.

Figure S3. Phylogenetic relationships based on whole genome sequencing of *Alloteropsis* accessions.

Figure S4. Phylogenetic relationships based on a sub-set of the whole genome sequencing of *Alloteropsis* accessions.

Figure S5. Assignment of *A. semialata* individuals to genetic clusters based on a sub-set of the aligned reads from the whole genome sequencing.

Figure S6. Percentage of heterozygous sites for each accession

Figure S7. Results of PCR amplification of *ppc-IP3* and *pck-IP1* in *Alloteropsis*.

Figure S8. Phylogeny of *pck-1P1* in *Alloteropsis.*

Figure S9. Divergence times for different nodes estimated from vertically-inherited and laterally-acquired genes.

Figure S10. Phylogeny of *ppc-1P3* in *Alloteropsis.*

Table S1. Sample and sequencing information.

Table S2. Alignment statistics of the *Alloteropsis* genome-skimming data to the *Setaria* reference genome.

Table S3. Primer pairs for amplification of genes copies of phosphoenolpyruvate carboxylase (*ppc*) and phosphoenolpyruvate carboxykinase (*pck*).

Supplementary Information 1. Scripts used to genotype the samples and produce a phylip file and an input file for Structure.

Supplementary Information 2. Scripts used to resample a subset of reads.

Supplementary Information 3. Perl scripts used identify and retrieve reads corresponding to different *pck* and *ppc* gene lineages.

Supplementary Figures and Tables

Figure S1. Distribution of the 170,629 called SNPs. Icons between each figure represents nine chromosomes, with the length and position of the centromere based on Bennetzen, *et al*. 2012. Figures above each chromosome icon represent all 170,629 called SNPs, and figures below each chromosome icon represent the 2,607 SNPs used for population structure analysis.

Figure S2. Phylogenetic relationships based on complete chloroplast genomes from Alloteropsis. This tree was obtained through Bayesian inference. Branch lengths are proportional to estimated divergence times and bootstrap support values are indicated near branches. Photosynthetic types are indicated with symbols at the tips. The main clades are delimited on the right. Bootstrap support values are shown near branches.

Figure S3. Phylogenetic relationships based on whole genome sequencing of *Alloteropsis* accessions. Bootstrap support values are indicated near branches. Colours of branches are based on groups of individuals as determined in the cluster analysis (Fig. 3), with branches leading to accessions with mixed genetic background in black. Photosynthetic types are indicated with symbols at the tips. Bootstrap support values are shown near branches.

Figure S4. Phylogenetic relationships based on a sub-set of the whole genome sequencing of *Alloteropsis* accessions. Bootstrap support values are indicated near branches. Colours of branches are based on groups of individuals as determined in the cluster analysis (Fig. 3), with branches leading to accessions with mixed genetic background in black. Photosynthetic types are indicated with symbols at the tips. Bootstrap support values are shown near branches.

Figure S5. Assignment of *A. semialata* individuals to genetic clusters based on a sub-set of the aligned reads from the whole genome sequencing. **A.** Assignment of each individual to the different clusters (for *K* 2-4**), B.** Mean likelihood (± s.d.) over 10 runs for each *K* value (1-10), and **C.** |L´´(*K*)|/s.d. as calculated according to Evanno, et al. (2005).

Figure S6. Percentage of heterozygous sites for each accession based on a sub-set of the aligned reads from the whole genome.

Figure S7. Results of PCR amplification of *ppc-IP3* and *pck-IP1* in *Alloteropsis*. The same or closely related individuals were used for PCR amplification and for genome skimming. A.cim

closely related to RCH20, A.ang closely related to 3C and Ang1, MTP5 and LSU4 closely related to RSA1 and RSA2 and L04A, L01B, L02G, and TW1 individuals from the same population as TAN1, TAN2, TAN4, and TPE1 respectively.

Figure S8. Phylogeny of *pck-1P1* in *Alloteropsis.* Laterally acquired gene from *Cenchrus* species is indicated (*pck-1P1_LGT:C*). Accessions in blue, green, yellow and red belong to clades I, II, III and IV, respectively, as they are represented in Fig. S3. Asterisks indicate gene sequences obtained from PCR-Sanger sequencing approach. Bootstrap support values are shown near branches when greater than 50.

Figure S9. Divergence times for different nodes estimated from vertically-inherited and laterally-acquired genes. A. The posterior distributions of times to the last common ancestor of *A. semialata* and *A. cimicina* is shown for the three genes resent in both species, in million years (Ma). B. The posterior distributions of times to the last common ancestor of *A. semialata* and *A. angusta* is shown for the three genes resent in both species, in million years (Ma).

Figure S10. Phylogeny of *ppc-1P3* in *Alloteropsis.* Laterally acquired genes from Andropogonae (*ppc-1P3_LGT:A*), *Setaria palmifolia* complex (*ppc-1P3_LGT:C*) and Melinidinae (*ppc-1P3_LGT:C*) are delimited on the right. Accessions in blue, green, yellow and red belong to clades I, II, III and IV, respectively, as they are represented in Fig. S3. Asterisks indicate gene sequences obtained from PCR-Sanger sequencing approach. Bootstrap support values are shown near branches when greater than 50.

Fig. S1

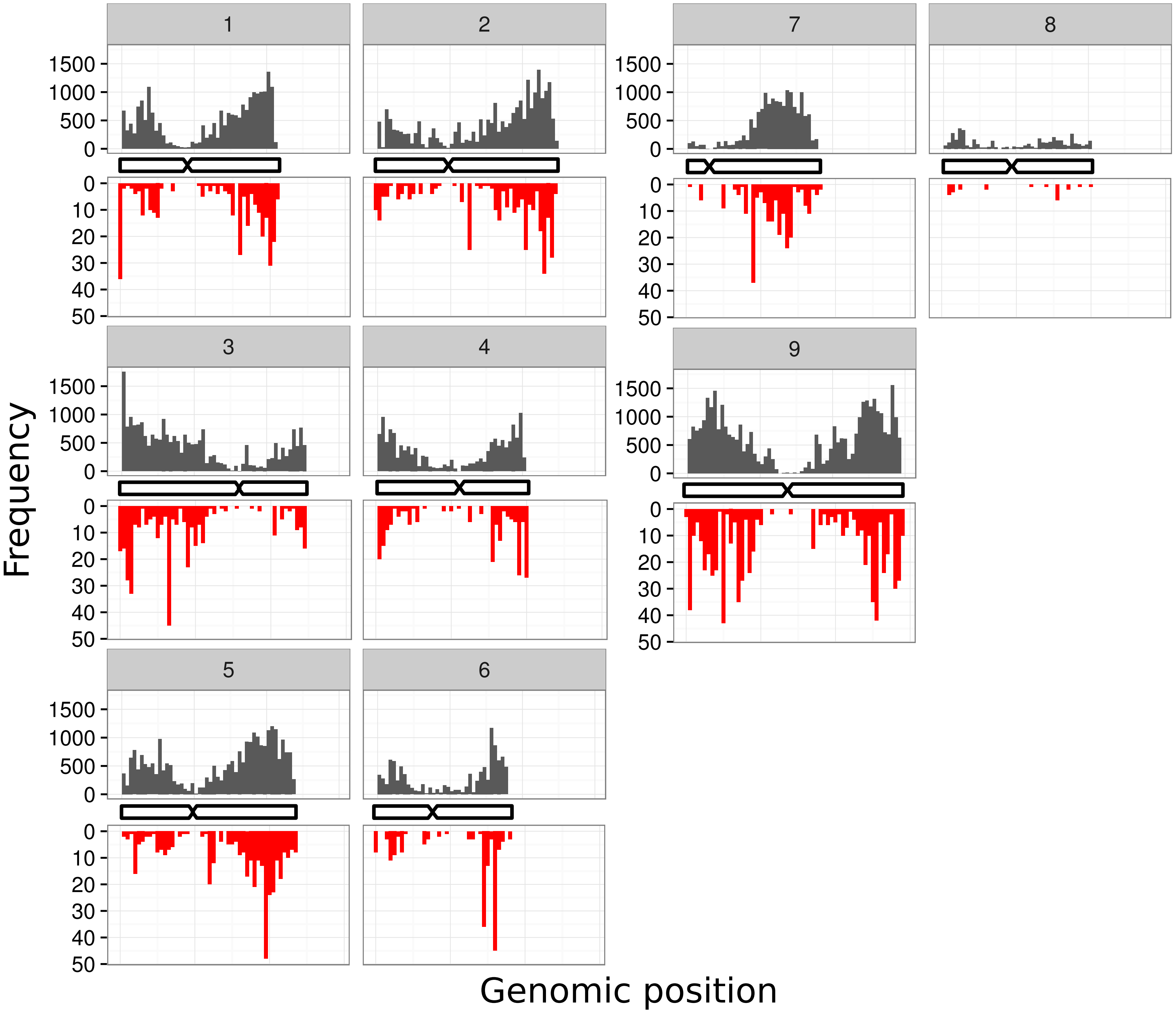

1
2
7
8
3
4
9
5
6
1500
1000
500
0
10
20
30
40
50
Frequency
Genomic position

Fig. S2

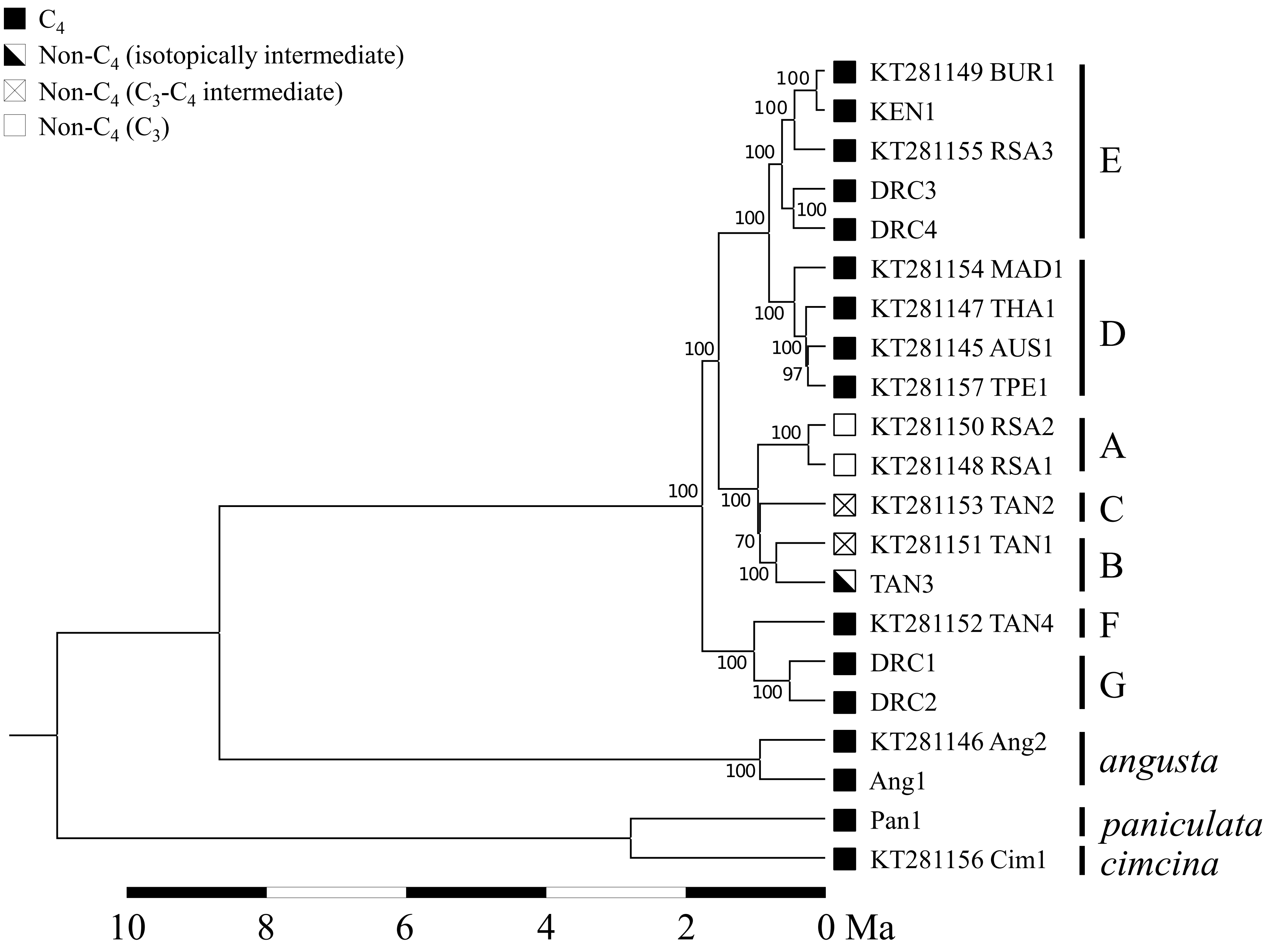

C4
Non-C4 (isotopically intermediate)
Non-C4 (C3-C4 intermediate)
Non-C4 (C3)
KT281149 BUR1
KEN1
KT281155 RSA3
DRC3
DRC4
KT281154 MAD1
KT281147 THA1
KT281145 AUS1
KT281157 TPE1
KT281150 RSA2
KT281148 RSA1
KT281153 TAN2
KT281151 TAN1
TAN3
KT281152 TAN4
DRC1
DRC2
KT281146 Ang2
Ang1
Pan1
KT281156 Cim1
E
D
A
C
B
F
G
angusta
paniculata
cimcina
100
97
70
10
8
6
4
2
0 Ma

Fig. S3

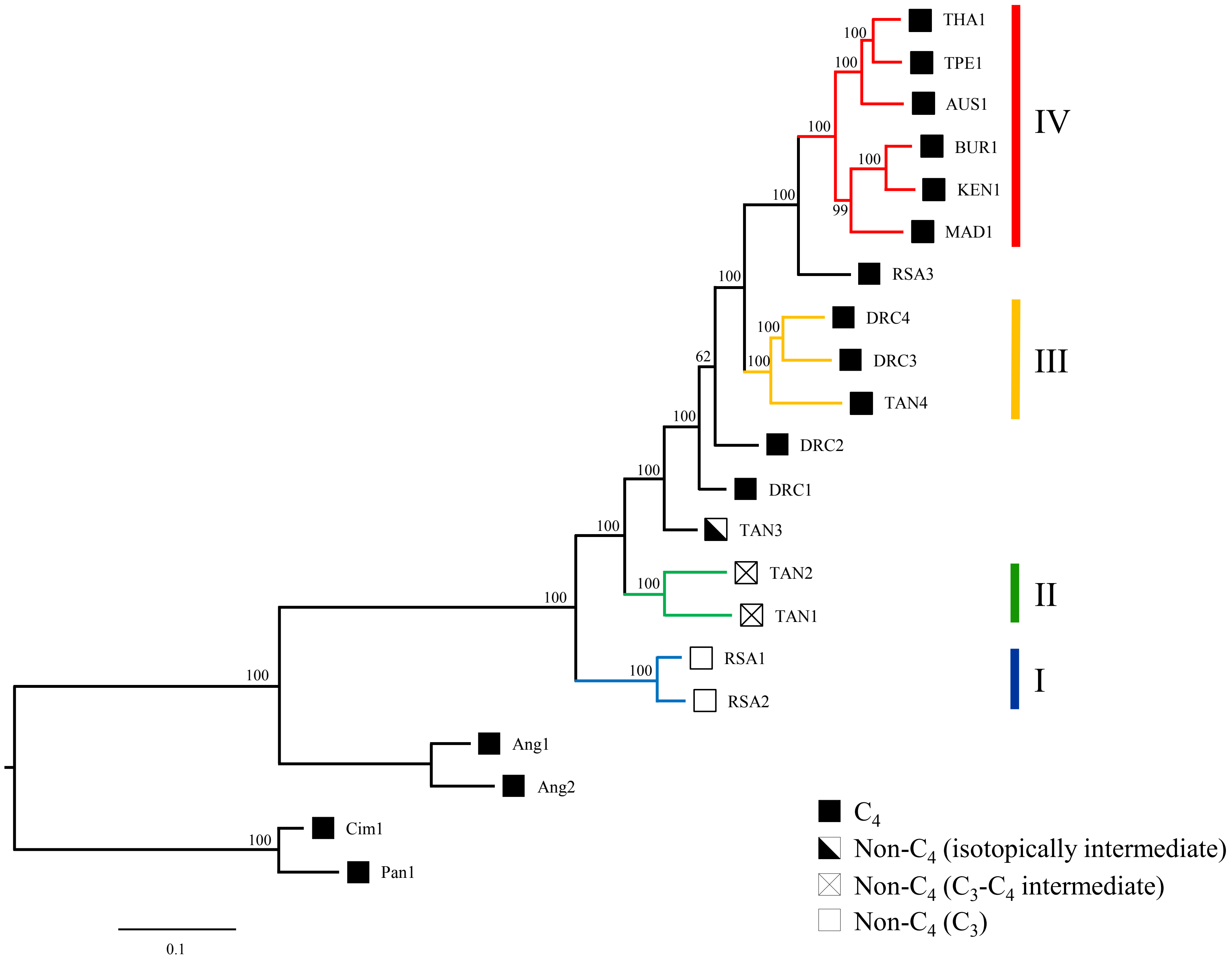

THA1
TPE1
AUS1
BUR1
KEN1
MAD1
RSA3
DRC4
DRC3
TAN4
DRC2
DRC1
TAN3
TAN2
TAN1
RSA1
RSA2
Ang1
Ang2
Cim1
Pan1
100
99
62
IV
III
II
I
0.1
C_4
Non-C_4 (isotopically intermediate)
Non-C_4 (C_3-C_4 intermediate)
Non-C_4 (C_3)

Fig. S4

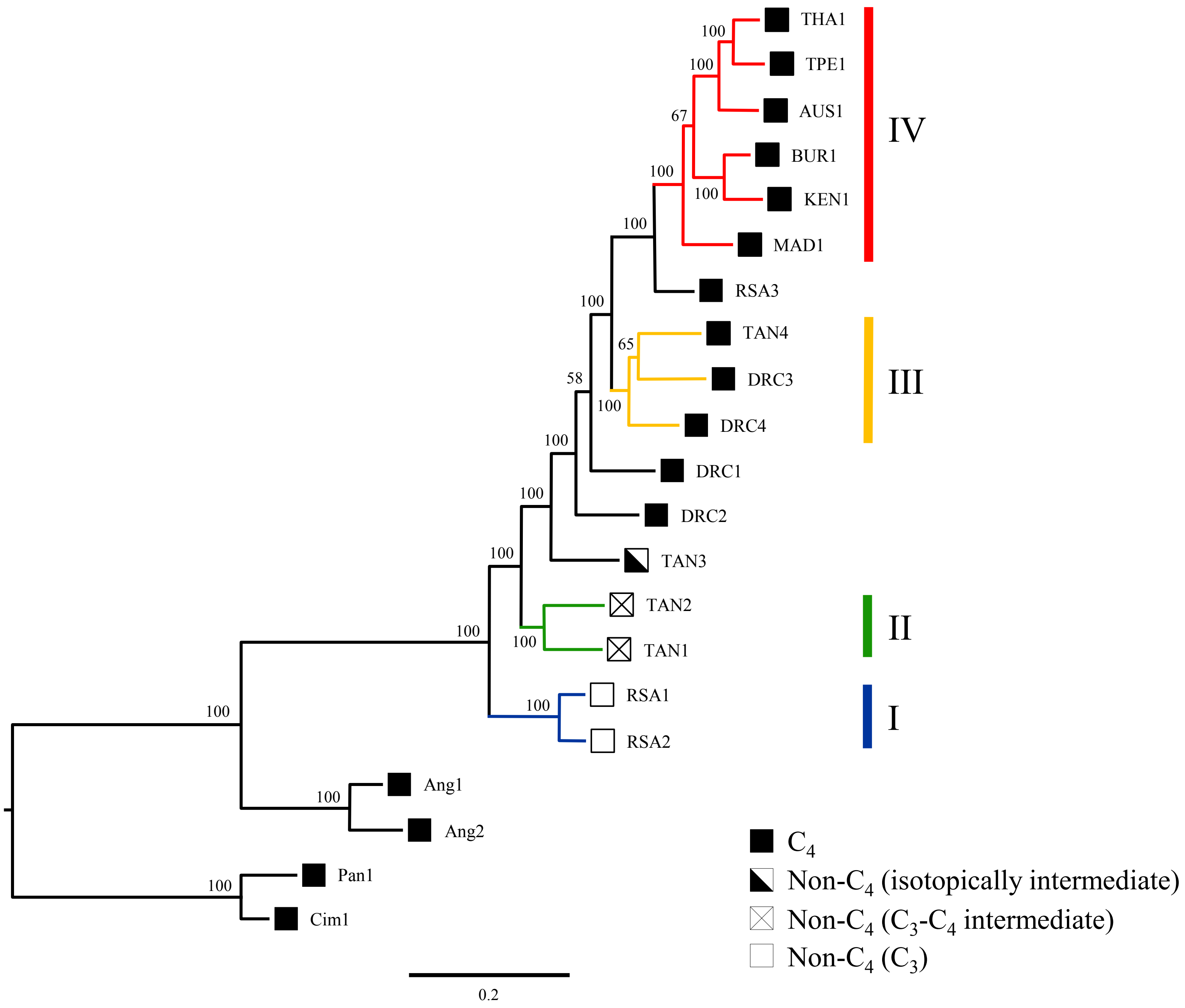
THA1
TPE1
AUS1
BUR1
KEN1
MAD1
RSA3
TAN4
DRC3
DRC4
DRC1
DRC2
TAN3
TAN2
TAN1
RSA1
RSA2
Ang1
Ang2
Pan1
Cim1
IV
III
II
I
100
67
65
58
0.2
C_4
Non-C_4 (isotopically intermediate)
Non-C_4 (C_3-C_4 intermediate)
Non-C_4 (C_3)

Fig. S5

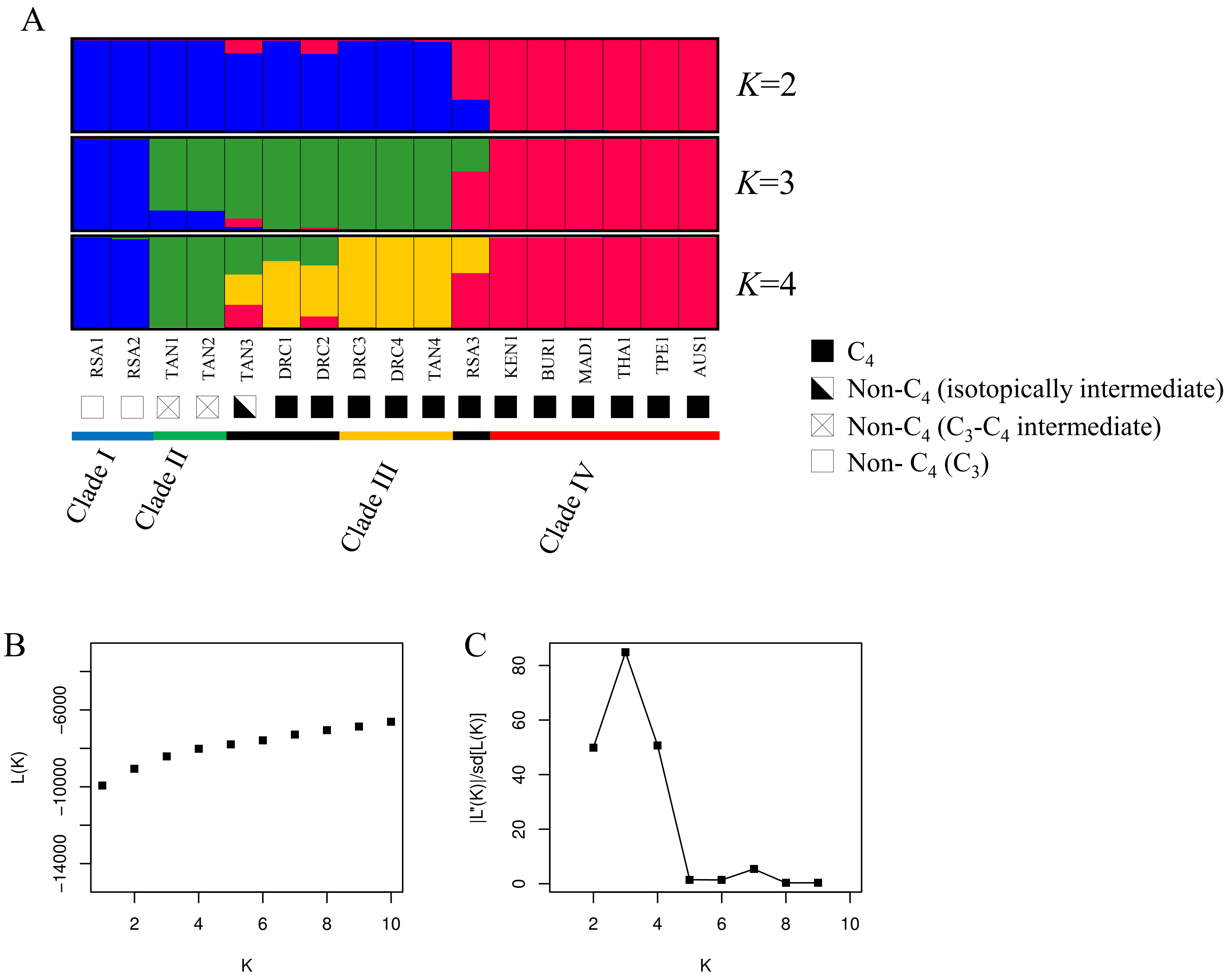

A
K=2
K=3
K=4
RSA1
RSA2
TAN1
TAN2
TAN3
DRC1
DRC2
DRC3
DRC4
TAN4
RSA3
KEN1
BUR1
MAD1
THA1
TPE1
AUS1
C_4
Non-C_4 (isotopically intermediate)
Non-C_4 (C_3-C_4 intermediate)
Non- C_4 (C_3)
Clade I
Clade II
Clade III
Clade IV
B
L(K)
−14000
−10000
−6000
2
4
6
8
10
K
C
|L"(K)|/sd[L(K)]
0
20
40
60
80
2
4
6
8
10
K

Fig. S6

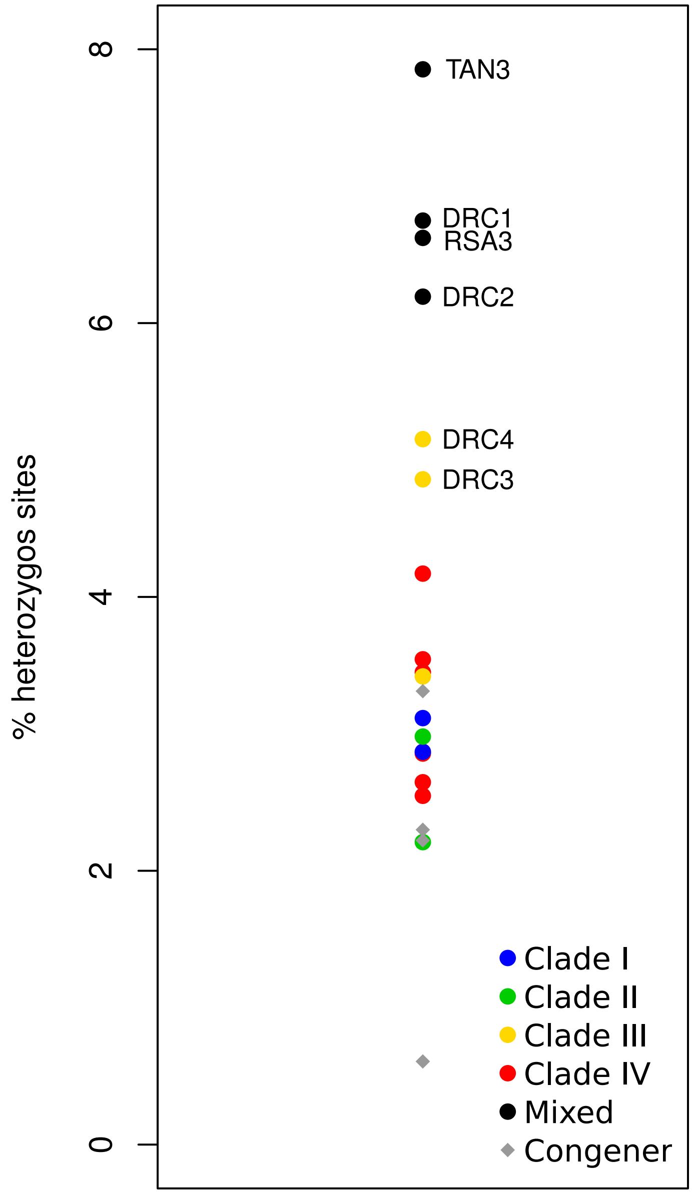

% heterozygos sites
0
2
4
6
8
TAN3
DRC1
RSA3
DRC2
DRC4
DRC3
Clade I
Clade II
Clade III
Clade IV
Mixed
Congener

Fig. S7

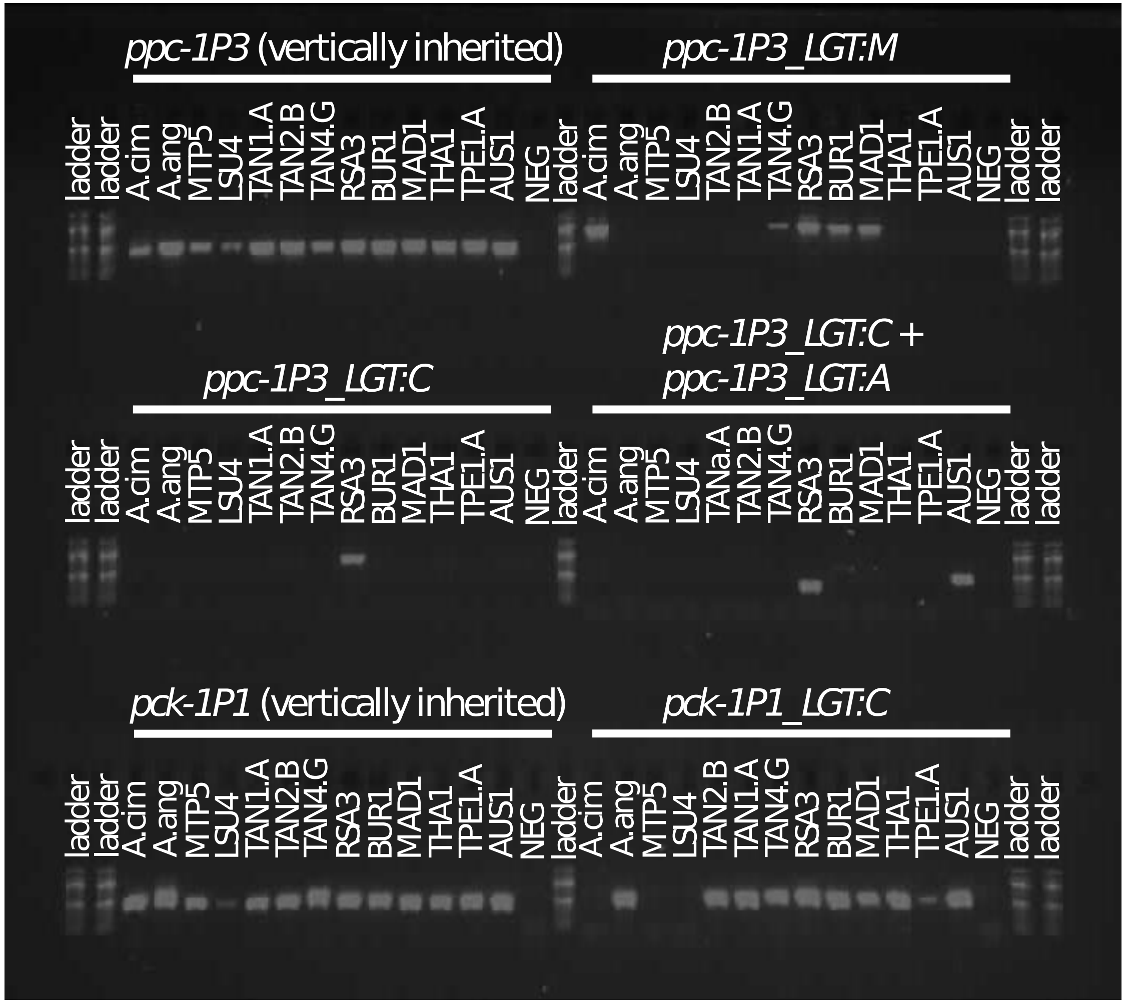
ppc-1P3 (vertically inherited)
ppc-1P3_LGT:M
ladder ladder A.cim A.ang MTP5 LSU4 TAN1.A TAN2.B TAN4.G RSA3 BUR1 MAD1 THA1 TPE1.A AUS1 NEG ladder A.cim A.ang MTP5 LSU4 TAN2.B TAN1.A TAN4.G RSA3 BUR1 MAD1 THA1 TPE1.A AUS1 NEG ladder ladder
ppc-1P3_LGT:C
ppc-1P3_LGT:C +
ppc-1P3_LGT:A
ladder ladder A.cim A.ang MTP5 LSU4 TAN1.A TAN2.B TAN4.G RSA3 BUR1 MAD1 THA1 TPE1.A AUS1 NEG ladder A.cim A.ang MTP5 LSU4 TANa.A TAN2.B TAN4.G RSA3 BUR1 MAD1 THA1 TPE1.A AUS1 NEG ladder ladder
pck-1P1 (vertically inherited)
pck-1P1_LGT:C
ladder ladder A.cim A.ang MTP5 LSU4 TAN1.A TAN2.B TAN4.G RSA3 BUR1 MAD1 THA1 TPE1.A AUS1 NEG ladder A.cim A.ang MTP5 LSU4 TAN2.B TAN1.A TAN4.G RSA3 BUR1 MAD1 THA1 TPE1.A AUS1 NEG ladder ladder

Fig. S8

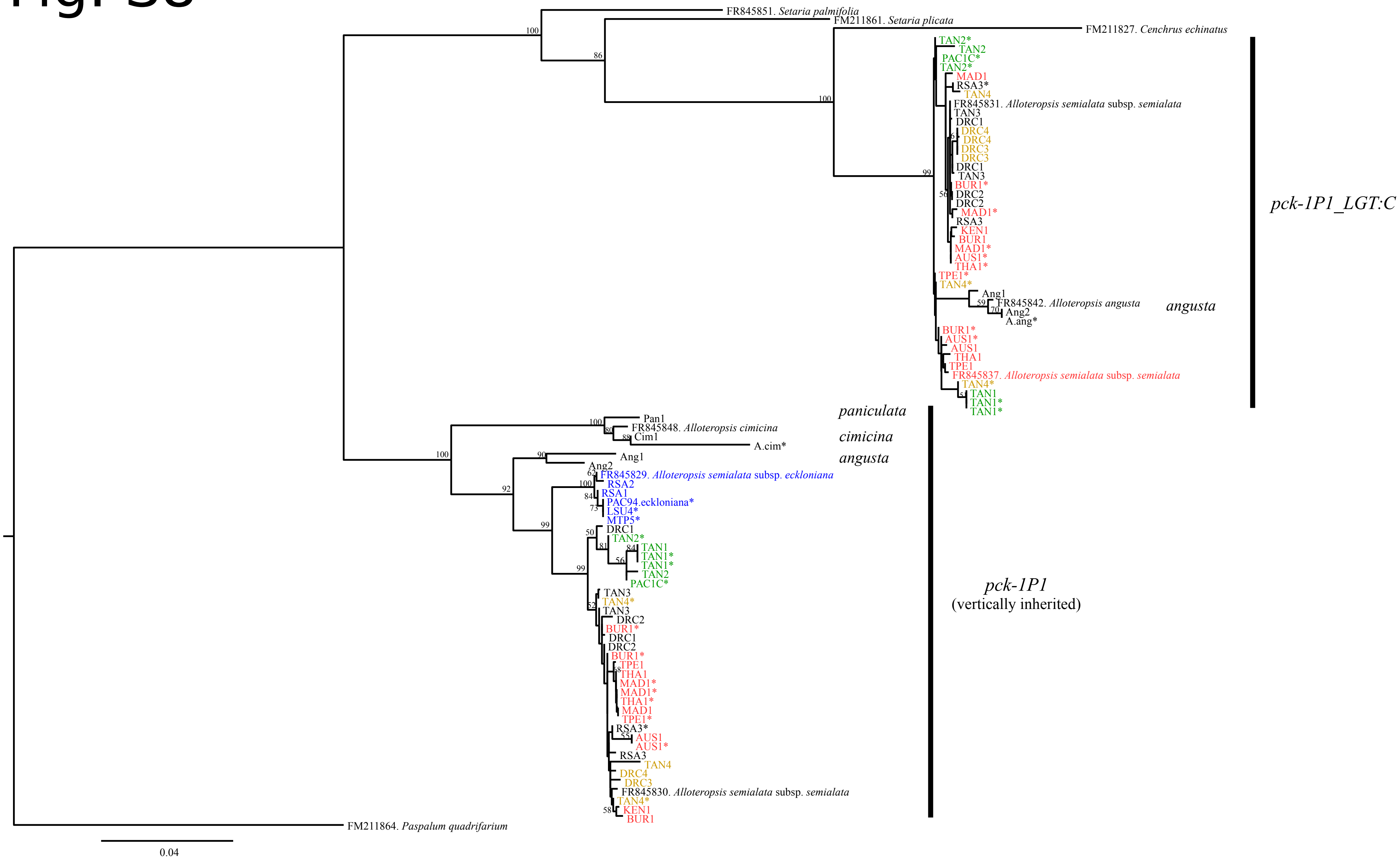

FR845851. Setaria palmifolia
FM211861. Setaria plicata
FM211827. Cenchrus echinatus
FR845831. Alloteropsis semialata subsp. semialata
FR845842. Alloteropsis angusta
FR845837. Alloteropsis semialata subsp. semialata
angusta
pck-1P1_LGT:C
paniculata
cimicina
angusta
FR845848. Alloteropsis cimicina
FR845829. Alloteropsis semialata subsp. eckloniana
PAC94.ekcloniana*
pck-1P1
(vertically inherited)
FR845830. Alloteropsis semialata subsp. semialata
FM211864. Paspalum quadrifarium
0.04

Fig. S9

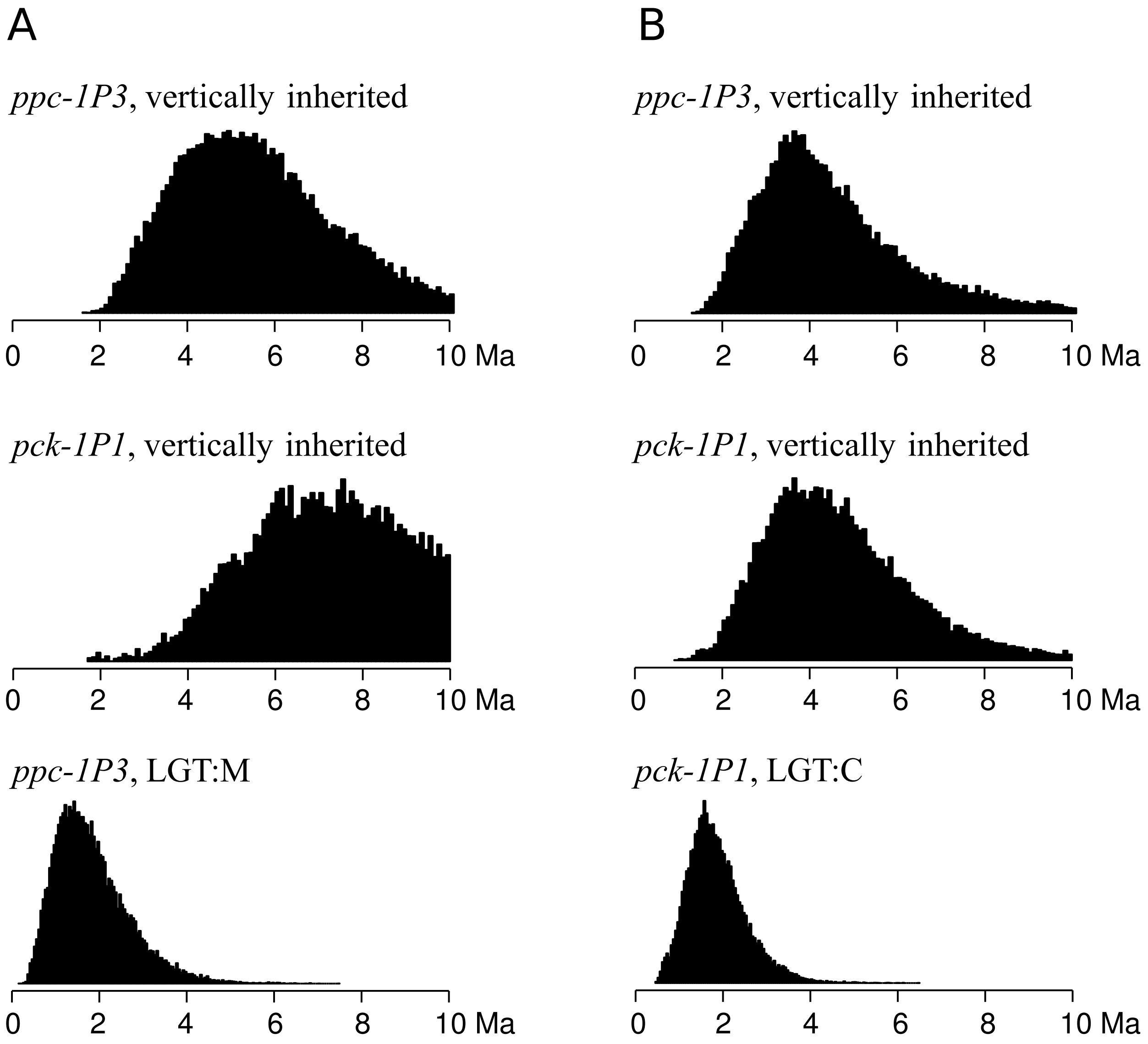
A
B
ppc-1P3, vertically inherited
pck-1P1, vertically inherited
ppc-1P3, LGT:M
pck-1P1, LGT:C
0
2
4
6
8
10 Ma

Fig. S10

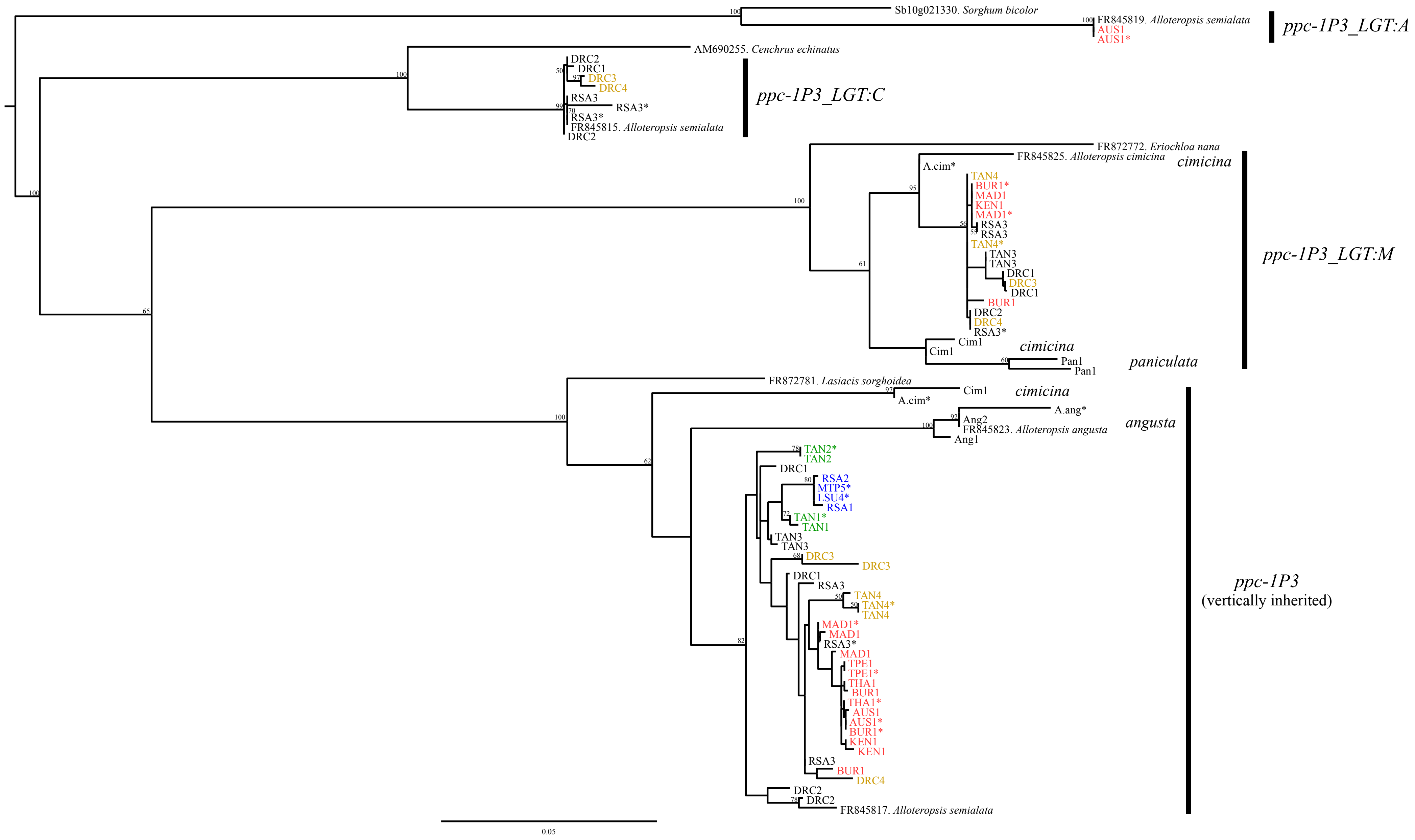
Sb10g021330. Sorghum bicolor
FR845819. Alloteropsis semialata
AUS1
AUS1*
ppc-1P3_LGT:A
AM690255. Cenchrus echinatus
DRC2
DRC1
DRC3
DRC4
RSA3
RSA3*
RSA3*
FR845815. Alloteropsis semialata
DRC2
ppc-1P3_LGT:C
FR872772. Eriochloa nana
FR845825. Alloteropsis cimicina
cimicina
A.cim*
TAN4
BUR1*
MAD1
KEN1
MAD1*
RSA3
RSA3
TAN4*
TAN3
TAN3
DRC1
DRC3
DRC1
BUR1
DRC2
DRC4
RSA3*
Cim1
Cim1
cimicina
Pan1
Pan1
paniculata
ppc-1P3_LGT:M
FR872781. Lasiacis sorghoidea
Cim1
A.cim*
cimicina
A.ang*
Ang2
FR845823. Alloteropsis angusta
Ang1
angusta
TAN2*
TAN2
DRC1
RSA2
MTP5*
LSU4*
RSA1
TAN1*
TAN1
TAN3
TAN3
DRC3
DRC3
DRC1
RSA3
TAN4
TAN4*
TAN4
MAD1*
MAD1
RSA3*
MAD1
TPE1
TPE1*
THA1
BUR1
THA1*
AUS1
AUS1*
BUR1*
KEN1
KEN1
RSA3
BUR1
DRC4
DRC2
DRC2
FR845817. Alloteropsis semialata
ppc-1P3
(vertically inherited)
0.05

Table S1. Sample and sequencing information.

ID	Sample name	Species	Genome size (Gb/1Cx[2])/ploidy	PT[3] [$\delta^{13}C$]	Country	Voucher	Year	Latitude	Longitude	Sequencer	Read length	Batch[7]
Cim1	RCH20	*A. cimicina*	-	C_4 [-]	Madagascar	Hall 20 (K)	2011	-18.77	46.87	HiSeq 2500	100	2
Pan1[1]	MSV627	*A. paniculata*	-	C_4[4] [-]	Madagascar	Vorontsova 627 (K)	2011	-18.77	46.87	HiSeq 2500	101	6
Ang1[1]	Ang1	*A. angusta*	-	C_4 [-10.20]	DRC	Pauwels 1182 (BR)	1959	-4.04	21.76	HiSeq 3000	150	5
Ang2	3C	*A. angusta*	0.97/2n	C_4 [-]	Uganda	Namaganda & Wanyana 3C (MHU)	2009	-0.36	31.87	HiSeq 2500	100	2
RSA1	BL	*A. semialata*	-	Non-C_4 [-28.00[5]]	South Africa	Lundgren & Ripley 11 (SHD)	2012	-29.71	29.96	HiSeq 2500	100	1
RSA2	JM	*A. semialata*	0.90/2n	Non-C_4 [-26.80[5]]	South Africa	Ripley 1 (SHD)	2012	-33.32	26.44	HiSeq 2500	100	1
TAN1	L04C	*A. semialata*	1.10/2n	Non-C_4 [-23.10[5,6]]	Tanzania	Lundgren & Christin 4 (SHD)	2014	-8.51	35.17	HiSeq 2500	100	2
TAN2	L01A	*A. semialata*	0.94/2n	Non-C_4 [-26.30[5]]	Tanzania	Lundgren & Christin 1 (SHD)	2014	-5.63	32.69	HiSeq 2500	100	2
TAN3[1]	39688	*A. semialata*	-	Non-C_4 [-18.60[5]]	Tanzania	Ruffo & Kisena 2806 (K)	1987	-7.87	31.67	HiSeq 2500	125	3
DRC1[1]	Asem4	*A. semialata*	-	C_4 [-10.75]	DRC	Kisimba & Malaisse 438 (BR)	2006	-10.36	26.08	HiSeq 3000	150	5
DRC2[1]	Asem2	*A. semialata*	-	C_4 [-11.22]	DRC	Lefebwe et al. 84 (BR)	1973	-10.42	26.18	HiSeq 3000	150	4
DRC3[1]	31768	*A. semialata*	-	C_4 [-10.60[5]]	DRC	Poelman 92 (K)	1961	-11.64	27.48	HiSeq 2500	125	3
DRC4[1]	Asem3	*A. semialata*	-	C_4 [-10.70]	DRC	Bulaimu 743 (BR)	1973	-11.64	27.48	HiSeq 3000	150	5
TAN4	L02O	*A. semialata*	1.01/2n	C_4 [-11.40[5]]	Tanzania	Lundgren & Christin 2 (SHD)	2014	-9.04	32.48	HiSeq 2500	100	2
RSA3	MD	*A. semialata*	0.87/6n	C_4 [-12.70[5,6]]	South Africa	Ibrahim 20 (SHD)	2004	-25.76	29.47	HiSeq 2500	100	1
KEN1[1]	AB3722	*A. semialata*	-	C_4 [-11.71[5]]	Kenya	Bogdan 3722 (EA)	1953	-0.02	37.91	HiSeq 2500	125	3
BUR1	Bur	*A. semialata*	0.98/2n	C_4 [-11.30[5]]	Burkina Faso	Sanou BUR-734	2009	10.85	-4.82	HiSeq 2500	100	1
MAD1	Ma	*A. semialata*	1.03/2n	C_4 [-11.80[5]]	Madagascar	Vorontsova 919 (K)	2013	-15.67	46.37	HiSeq 2500	100	1
THA1	ATSS837	*A. semialata*	-	C_4 [-12.20[5]]	Thailand	AT & SS 837 (TCD)	2007	18.41	100.33	HiSeq 2500	100	2
TPE1	TW3	*A. semialata*	0.94/2n	C_4 [-14.60[5,6]]	Taiwan	-	2014	24.47	120.72	HiSeq 2500	100	2
AUS1	Aus	*A. semialata*	1.1/2n	C_4 [-12.10[5]]	Australia	AusTRCF 322458 0167	2005	-19.62	146.96	HiSeq 2500	100	1

[1]newly sequenced sample; [2] 1Cx: monoploid genome size (DNA content per basic chromosome set; Greilhuber *et al.* 2005); [3]PT – photosynthetic type; [4] based on leaf anatomy (Christin *et al*. 2013b); [5]from Lundgren *et al*. 2015; [6]measured on a different accession from the same population; [7] Samples with the same number were sequenced together.

Table S2. Alignment statistics of the *Alloteropsis* genome-skimming data to the *Setaria* reference genome.

Sample	Filtered pair-end reads	Genome-wide coverage[1]	Total number of reads aligned	Pair-end reads concordantly aligned exactly one time	Total number of reads aligned to CDS	Individual coverage cut-off	Positions genotyped (% missing data)[2]	Positions genotyped in sub-set (% missing data)[3]
**Cim1**	20,415,006	1.86	1,176,915 (5.76%)	425,408 (2.08%)	438,307 (2.15%)	5	144,430 (15.4)	16,758 (26.6)
**Pan1**	7,740,126	0.70	792,297 (10.23%)	197,804 (2.56%)	227,351 (2.94%)	2	49,255 (71.1)	8,725 (61.7)
**Ang1**	14,343,018	1.96	579,842 (4.04%)	246,072 (1.72%)	175,529 (1.22%)	5	103,946 (39,1)	13,132 (42.4)
**Ang2**	18,067,598	1.86	831,340 (4.60%)	266,482 (1.47%)	295,435 (1.64%)	5	98,565 (42.2)	13,010 (43.0)
**RSA1**	14,326,452	1.30	751,861 (5.25%)	182,288 (1.27%)	247,120 (1.72%)	4	92,017 (46.1)	13,354 (41.4)
**RSA2**	12,069,794	1.34	523,241 (4.34%)	125,338 (1.04%)	169,927 (1.41%)	3	78,975 (53.7)	13,558 (40.6)
**TAN1**	18,821,504	1.71	1,404,829 (7.46%)	476,264 (2.53%)	397,122 (2.11%)	5	120,329 (29.5)	12,086 (47.0)
**TAN2**	20,041,562	2.13	1,532,343 (7.65%)	510,090 (2.55%)	412,690 (2.06%)	5	120,289 (29.5)	11,208 (50.9)
**TAN3**	33,537,244	3.81	1,856,861 (5.54%)	798,550 (2.38%)	570,630 (1.70%)	10	149,328 (12.5)	14,592 (36.0)
**DRC1**	33,118,910	4.52	1,997,844 (6.03%)	754,382 (2.28%)	590,736 (1.78%)	12	155,930 (8.6)	14,210 (37.8)
**DRC2**	23,353,806	3.18	1,291,044 (5.96%)	536,120 (2.30%)	404,876 (1.73%)	9	145,775 (14.6)	14,193 (37.8)
**DRC3**	28,530,034	3.24	2,595,576 (9.10%)	1,078,654 (3.73%)	763,728 (2.68%)	9	139,147 (18.5)	9,446 (58.6)
**DRC4**	14,480,696	1.97	908,170 (6.27%)	364,370 (2.52%)	260,236 (1.80%)	5	124,770 (26.9)	12,945 (43.3)
**TAN4**	18,395,178	1.82	1,233,515 (6.71%)	410,334 (2.23%)	349,951 (1.90%)	5	120,231 (29.5)	12,955 (43.2)
**RSA3**	13,396,464	1.54	611,585 (4.57%)	167,612 (1.25%)	217,359 (1.62%)	3	100,694 (41.0)	16,082 (29.5)
**KEN1**	24,717,950	2.80	1,703,210 (6.89%)	663,054 (2.68%)	418,286 (1.69%)	8	104,045 (39.0)	8,683 (62.0)
**BUR1**	13,103,476	1.33	578,025 (4.41%)	148,298 (1.13%)	173,457 (1.36%)	3	93,650 (45.1)	16,114 (29.4)
**MAD1**	16,120,906	1.57	836,086 (5.19%)	229,338 (1.42%)	245,711 (1.52%)	4	102,932 (39.7)	14,360 (37.1)
**THA1**	16,557,636	1.50	1,010,043 (6.10%)	313,698 (1.89%)	279,155 (1.69%)	4	118,067 (30.8)	14,089 (38.3)
**TPE1**	15,505,844	1.65	1,228,659 (7.92%)	423,360 (2.73%)	345,175 (2.23%)	4	109,410 (35.9)	11,449 (49.9)
**AUS1**	11,246,150	1.02	473,380 (4.21%)	118,012 (1.05%)	149,553 (1.33%)	3	79,564 (53.4)	14,446 (36.7)

[1] Estimated based on a genome sizes given in Table S1; for accessions with unknown genomes size the largest value (1.1 Gb) was used; [2] Total of 170,629 across all accessions; [3] Total of 22,821 across all accessions

Table S3. Primers used to amplify the different *ppc* and *pck* genes.

**Gene copy**	**Forward primer**	**Reverse primer**	**Annealing temperature (ºC)**	**Extension time (s)**
ppc-1P3_native	5’-GCTTCCGCACGCTGCAGCGG-3’	5’-CTCTGAGCACCTGGATGTTCC-3’	57	60
ppc-1P3_LGT:M	5’-AGCGTGAGTGCAAAGTGGCAG-3’	5’-GTGACCCTGAARAAKGGCCAC-3’	57	60
ppc-1P3_LGT:C	5’-GCGAGTGCCACATAAAGGAG-3’	5’-GTGACCCTGAARAAKGGCCAC-3’	57	60
ppc-1P3_LGT:A +ppc-1P3_LGT:C	5’-CGCTCCGTGGTCGTSAAGG-3’	5’-CAGGGTGACCCTGAAGAATG-3’	54	30
pck-1P1_native	5’-TGTCGACGGATCACAATAGGC-3’	5’-TACTCGATCGGGTACGCAGCC-3’	57	60
pck-1P1_LGT:C	5’-GACGACGCTGTCGACGGATCC-3’	5’-ACGGGTGTTCTCTGCATGCAG-3’	57	60
